# Identification of Modulators of HIV-1 Proviral Transcription from a Library of FDA-Approved Pharmaceuticals

**DOI:** 10.3390/v12101067

**Published:** 2020-09-23

**Authors:** Gavin C. Sampey, Sergey Iordanskiy, Michelle L. Pleet, Catherine DeMarino, Fabio Romerio, Renaud Mahieux, Fatah Kashanchi

**Affiliations:** 1 Laboratory of Molecular Virology, George Mason University, Manassas, VA 20110, USA; gavin.sampey@gmail.com (G.C.S.); siord6@GMAIL.COM (S.I.); mlpleet@gmail.com (M.L.P.); cdemarin@gmu.edu (C.D.); 2 Institute of Human Virology, University of Maryland School of Medicine, Baltimore, MD 21201, USA; fromeri2@jhmi.edu; 3 Equipe Oncogenèse Rétrovirale, Equipe Labellisée Ligue Nationale Contre le Cancer, Centre International de Recherche en Infectiologie, INSERM U1111-CNRS UMR5308, 69372 Lyon, France; renaud.mahieux@ens-lyon.fr

**Keywords:** HIV, latency, transcription, activator, inhibitor, febuxostat, eltrombopag, resveratrol, mycophenolate

## Abstract

Human immunodeficiency virus 1 (HIV-1) is the most prevalent human retrovirus. Recent data show that 34 million people are living with HIV-1 worldwide. HIV-1 infections can lead to AIDS which still causes nearly 20,000 deaths annually in the USA alone. As this retrovirus leads to high morbidity and mortality conditions, more effective therapeutic regimens must be developed to treat these viral infections. A key target for intervention for which there are no current FDA-approved modulators is at the point of proviral transcription. One successful method for identifying novel therapeutics for treating infectious diseases is the repurposing of pharmaceuticals that are approved by the FDA for alternate indications. Major benefits of using FDA-approved drugs include the fact that the compounds have well established toxicity profiles, approved manufacturing processes, and immediate commercial availability to the patients. Here, we demonstrate that pharmaceuticals previously approved for other indications can be utilized to either activate or inhibit HIV-1 proviral transcription. Specifically, we found febuxostat, eltrombopag, and resveratrol to be activators of HIV-1 transcription, while mycophenolate was our lead inhibitor of HIV-1 transcription. Additionally, we observed that the infected cells of lymphoid and myeloid lineage responded differently to our lead transcriptional modulators. Finally, we demonstrated that the use of a multi-dose regimen allowed for enhanced activation with our transcriptional activators.

## 1. Introduction

Retroviruses are difficult-to-treat pathogens that integrate their viral genome into the host’s DNA and induce numerous disease states in those infected, many of which can be severely adverse. The most common retrovirus that is transmitted in humans is human immunodeficiency virus 1 (HIV-1). As of 2011, 34 million people were living with HIV-1 worldwide and an additional 2.5 million new HIV infections took place that year [1]. Furthermore, AIDS-related causes kill nearly 20,000 people in the USA per year and HIV-associated neurocognitive disorders (HAND) are diagnosed in up to 47% of patients despite the use of highly active antiretroviral therapy (HAART) [1,2]. Due to the persistence of this retroviral infection and the devastating pathogenic states it induces, it is critical that more effective treatment regimens be developed to battle this virus.

Despite decades of research [3], a sterilizing or even functional cure for HIV-1 has remained elusive. While current HAART regimens have greatly decreased the mortality rates of infected individuals [4,5], as well as reduced the requisite pill burden [6,7,8], numerous complications are still associated with long-term controlled infection. Some of the most commonly observed problems include the re-emergence of the virus due to poor protocol adherence or the evolution of drug-resistant strains [9,10,11,12], as well as cognitive impairment due to HAND [2,13]. The primary culprit for the inability of HAART to purge all infected cells is the reservoir of latently infected cells that predominantly consists of the long-lived resting memory CD4+ T cells [14,15,16], although numerous other cell types contribute to this latent population [17,18,19,20,21,22]. Additionally, a significant portion of the latent reservoir is also established in the lymphatic and central nervous system (CNS), increasing the complexity in treatment options due to the selective permeability of these organs, such as that afforded by the blood–brain barrier (BBB) [20,23]. Prior to reactivation, the latent viral pool within the quiescent memory CD4+ T cells is highly persistent in the host with a half-life of up to 44 months for patients on HAART and no detected recurrence of viremia [24]. The extended half-life of the latent pool is increased in part by occasional short-term relapses of viremia in patients on HAART [25]. Moreover, since current therapeutics only block the transmission of the virus and do not address the cells that already harbor the integrated provirus; the latent pool of infected cells can re-establish active viral spread whenever HAART treatment is ceased.

Current HAART regimens include six classes of drugs that target various steps in the HIV-1 life cycle and can be identified as one of the following: nucleoside/nucleotide reverse transcriptase inhibitors (NRTIs), non-nucleoside reverse transcriptase inhibitors (NNRTIs), protease inhibitors (PIs), integrase strand transfer inhibitors (INSTIs), fusion inhibitors (FIs), or CCR5 antagonists. A key target for intervention in HIV-1 infections for which there are no current inhibitors is at the point of proviral transcription. For efficient HIV-1 proviral transcription to take place, the viral transcriptional transactivating protein Tat is required. Certain cellular stimuli, including endotoxins, cytokines such as tumor necrosis factor-α (TNFα) and interleukin-1 (IL-1), as well as the activation of the T cell receptor (TCR) by co-stimulation of CD3 and CD28, can allow for initial rounds of transcription via NF-κB transcriptional activation [26,27,28,29]. For monocytes and macrophages containing the latent virus, priming with interferon-γ (INFγ) or IL-10 followed by additional stimulation with other cytokines or endotoxin can potentiate viral expression [27,28]. After sufficient levels of the Tat are produced from the initial rounds of transcription, Tat-mediated transcription upregulates NF-κB-driven transcription to generate robust viral mRNA production by enhancing transcriptional initiation and elongation [29,30]. Mechanistically, Tat binds to the stem–loop structure of nascent proviral transcripts known as the transactivating response (TAR) element [31,32]. In addition to binding to TAR, Tat also recruits several transcription factors to the provirus promoter, of which the positive transcriptional elongation factor-b (P-TEFb) is of significant importance. Tat simultaneously binds TAR and P-TEFb, bringing the P-TEFb kinase complex into close proximity of the proximally paused RNA polymerase II (Pol II) and allowing it to phosphorylate numerous residues of the C-terminal domain (CTD) of the Pol II [29,30]. The hyperphosphorylated CTD then allows for the successful transcriptional elongation of Pol II and thereby, the enhanced production of full-length viral mRNA. The addition of an inhibitor of proviral transcription could compliment the current HAART regimens by acting upon a different molecular mechanism, thereby increasing the inhibition of HIV-1 replication in all viral reservoirs.

In addition to the current HIV-1 standard of care which involves the inhibition of viral spread, the pharmaceutical activation of latent pools in combination with current therapeutic HAART regimens has become a primary focus of the research community with regard to developing a sterilizing or functional cure. The major hurdle of any therapeutic regimen combining latent virus reactivation with current HAART is that every single latently infected cell must be eliminated, since, in theory, it only takes a few surviving latent cells to re-establish viremia. Therefore, any successful latency reversing agents (LRAs) need to penetrate all tissues harboring latent pools, including crossing the highly selective BBB, and be effective in all long-lived latent virus-harboring cellular sub-types, including quiescent memory CD4+ T cells and macrophages [14,15,16,22,33]. This broad efficacy across tissues and cell types has proven to be problematic even with the current HAART, as the various drugs in the cocktail have shown differential uptake into the lymphatic tissue of the lymph nodes, ileum, and rectum [23]. Despite these challenges, one highly researched clinical approach to viral reactivation is the use of histone deacetylase inhibitors (HDACis) to disrupt the highly ordered heterochromatin structure found in the 5′ long-terminal repeat (LTR) of a latent provirus. This method of viral replication has been examined in vitro since the mid-1990s [34]. More recently, the addition of HDACis to HAART has been tested in clinical trials with additional protocols underway. One study using valproic acid in combination with various antiviral regimens showed no significant reduction in infected resting memory CD4+ T cells [35], but despite this treatment failure, more potent HDACis are currently being investigated in human clinical trials for the eradication of latent pools [36]. However, the challenge of demonstrating efficacy across all latent virus-harboring cell types persists even with HDACis, as recent studies have shown them to downregulate viral production in macrophages [37]. Therefore, an alternate strategy to the use of broad non-specific HDACis for the reactivation of latent pools would be to upregulate Tat-mediated proviral transcription. A specific activator of Tat-driven transcription would be beneficial in that it would only impact the activity of the viral promoter and thereby reduce any off-target activation of cellular genes that may be toxic or oncogenic. Therefore, in examining the modulators of the Tat-mediated transcriptional pathway, both inhibitors and activators could harbor potential therapeutic value in the current strategies being pursued by the research community.

One successful method for identifying novel therapeutics for the treatment of infectious diseases is the repurposing of pharmaceutical compounds that are already approved by the FDA for alternate indications. To this end, the screening of large libraries of FDA-approved drugs has already been tested in the treatment of Ebola and other emerging infectious diseases [38,39,40], as well as in treating HIV-1 to a lesser extent. Some of the major benefits of utilizing FDA-approved drugs include the fact that the compounds have well established toxicity profiles in humans, approved manufacturing processes, and immediate commercial availability to the target patient populations. One potential downside to reusing FDA-approved drugs for alternate indications is the toxicity profiles of some of the compounds. For instance, a chemotherapeutic indicated for the treatment of a certain cancer type but that generates numerous adverse events (AEs), which are acceptable in terminal patients, would not likely be acceptable as a first-line antiretroviral in HIV-1-infected patients. This is due to the fact that the current standard of care in HAART causes few AEs and has high efficacy, yet despite this possible draw-back, these higher AE-generating drugs could be utilized as a rescue treatment in virally relapsed patients. Here, we screened a panel of 420 FDA-approved drugs to identify those that modulate the proviral transcription of HIV-1. While the active proviral transcription of HIV-1 depends upon Tat, it also requires the manipulation of different host molecular machinery, such as NF-κB. Therefore, it is expected that therapeutic compounds with varied mechanisms of action (MOA) will be identified that act upon the normal functioning of these two distinct retroviral transcriptional processes.

Here, we demonstrate that pharmaceuticals previously approved by the FDA for other indications can be utilized to either activate or inhibit Tat-mediated transcription in HIV-1 infections. The most potent proviral Tat transcriptional inhibitors, as well as activators of Tat-mediated transcription, were first identified using a model reporter and chronically infected cell lines. Then, the effectiveness of the top hits was tested on infected primary T cell and macrophage cultures. Lastly, the molecular MOAs for these activators and inhibitors were examined. During this study, we also observed that the lead modulators elicited differing responses from infected T cells and cells of myeloid lineage. Specifically, our lead activators of HIV-1-infected T cells and even our control activator SAHA did not produce significant increases in proviral transcription in infected cells of myeloid lineage. Moreover, our lead inhibitor in T cells, mycophenolate, actually increased HIV-1 transcription in our infected myeloid cells. Despite the differences in response between T cells and myeloid cells, we were able to generate enhanced and prolonged transcriptional activation from infected T cell lines with our lead activators by implementing a daily dosing regimen over the span of two to three days.

## 2. Materials and Methods

### 2.1. Cell Lines

The HIV-1 transcription reporter cell line TZM-bl was obtained through the NIH AIDS Reagent Program, Division of AIDS, NIAID, NIH: from Dr. John C. Kappes, Dr. Xiaoyun Wu and Tranzyme Inc. (Durham, NC, USA) [41,42,43,44,45]. TZM-bl cells are engineered HeLa cells that express CD4, CCR5, and CXCR4 and contain integrated reporter genes for firefly luciferase and β-galactosidase under the control of an HIV-1 long terminal repeat (LTR) [44]. This adherent reporter cell line was cultured in Dulbecco’s modified Eagle medium (DMEM) medium supplemented with 10% heat-inactivated fetal bovine serum (FBS), 2 mM l-glutamine, 100 U/mL penicillin, and 100 µg/mL streptomycin. An alternate HIV-1 transcription reporter cell line, Jurkat E4, was also tested with our top potential activators. The Jurkat E4 cell line harbors a mutated latent HIV-1 genome with the Gag gene deleted and the Nef gene replaced by the short half-life d2EGFP reporter gene [46]. In addition to the Jurkat E4 reporter cell line, the chronically HIV-1-infected suspension T-cell lines J1.1 and ACH2, as well as the promonocytic OM10.1 cell line, were cultured in RPMI-1640 medium supplemented with 10% heat-inactivated FBS, 2 mM l-glutamine, 100 U/mL penicillin, and 100 µg/mL streptomycin. The J1.1 cell line is a Jurkat E6.1 derivative chronically infected with HIV-1 (LAI strain) [47], while the ACH2 cell line was isolated from HIV-1 (LAV strain) infected A3.01 cells [48]. The OM10.1 cell line was derived from infected HL-60 promyelocytic cells [49]. ACH2, J1.1 and OM10.1 cells each contain a single integrated copy of the HIV-1 genome [48,49].

### 2.2. Isolation and Culture of Peripheral Blood Mononuclear Cells (PBMCs)

Four independent PBMCs (Precision For Medicine, Frederick, MD, USA) were cultured and activated with phytohemagglutinin and IL-2 for 72 h. Non-adherent cells were removed and further kept in media with PHA and IL-2 for another 48 h (activated T-cells). The adherent cells were treated with phorbol 12-myristate 13-acetate (PMA) for 4 days (macrophages) and then both T-cells and macrophages were infected with HIV-1 (89.6; multiplicity of infection = 1.0) and 3 days post infection, treated with combined antiretroviral therapy (cART) for 10 days. Cells were treated with various drug compounds post cART.

### 2.3. Transfections and Luciferase Assay for Initial Drug Screen

Prior to screening the FDA-approved drugs, the optimization of the fast-forward transfection conditions of pc-Tat into the TZM-bl cell line was carried out to ensure the reporter assay gave a Z-factor greater than 0.5. The Z-factor is a statistical measure that evaluates the signal-to-noise and signal-to-background of a given assay, and any assay with a Z-factor between 0.5 and 1.0 is considered excellent for high throughput screening. The Z-factor itself is defined as follows [50]:

Z-factor = 1 − ((3σ_s_ + 3σ_c_)/|µ_s_ − µ_c_|)

In this equation, σ_s_ and σ_c_ are the standard deviation of the positive control and background control, respectively, and µ_s_ and µ_c_ are the mean of the positive control and background control, respectively. For the optimized pc-Tat transfection of TZM-bl cells, the σ_s_ and σ_c_ were 3079 and 83.41 relative luminescence units (RLUs), respectively, and the µ_s_ and µ_c_ were 63,083 and 1775 RLUs, respectively, which yielded a Z-factor of 0.85 indicating its suitability for the drug screen. For the initial drug screen, TZM-bl cells were transfected using the optimized fast-forward transfection protocol. Specifically, pc-Tat (0.5 μg per reaction) and Attractene reagent (0.5 µL per reaction, Qiagen, Chatsworth, CA, USA) were mixed with DMEM without antibiotics and FBS (50 µL per reaction) to generate a master transfection mix. Then, 50 µL of the master transfection mix was added to each control and experimental well of a 96-well clear flat-bottom white tissue culture-treated plate (Corning, Corning, NY, USA) seeded with 5 × 10^4^ TZM-bl cells in 100 µL of complete DMEM medium. The transfected cells were incubated overnight before replacing the media with complete DMEM and treating the cells with the 420 drugs or controls (DMSO and flavopiridol) at a final concentration of 1 μM. The 420 FDA-approved drugs were distributed amongst six 96-well plates as our drug stocks at a concentration of 25 μM. Additionally, for each experimental transfection plate, four 0.01% DMSO-treated controls and four transcriptional inhibitor control wells treated with the P-TEFb inhibitor flavopiridol (100 nM) were run. Forty-eight hours post drug treatment, firefly luciferase activity was measured with the BrightGlo Luciferase Assay (Promega, Madison, WI, USA) following the manufacturer’s instructions and luminescence was read using an integration time of 0.5 s on a Promega Glomax Multi Detection System (Berthold Technologies, Oak Ridge, TN, USA).

For testing the effects of the FDA-approved drugs on the cytomegalovirus (CMV) promoter, the transfection of HeLa cells with a pc-Luc vector was carried out using a similar transfection protocol as the TZM-bl screen. Specifically, pc-Luc (0.25 μg per reaction) and Attractene reagent (0.5 µL per reaction) was mixed with DMEM without antibiotics and FBS (50 µL per reaction) to generate a master transfection mix. Then, 50 µL of the master transfection mixes were added to each control and experimental well of a 96-well white plate seeded with 5 × 10^4^ HeLa cells in 100 µL of complete DMEM medium. The transfected cells were incubated overnight before replacing the media with complete DMEM and treating the cells with the lead drug candidates from the initial screen or controls (DMSO and flavopiridol) at a final concentration of 1 μM. Forty-eight hours post drug treatment, firefly luciferase activity was measured with the BrightGlo Luciferase Assay and luminescence was read using an integration time of 0.5 s on a Promega Glomax Multi Detection System. The pc-Luc plasmid used in these transfections was a gift from William Kaelin (Addgene plasmid # 18964) [51].

In order to determine if the lead HIV-1 transcriptional modulators were dependent on or independent of Tat-mediated transcription, TZM-bl transfections with Tat mutants were conducted followed by treatment with lead activators and inhibitors. Specifically, transfections with pcFLAG-Tat (wt), pcFLAG-Tat (K41A) [52,53,54,55], and pcFLAG-Tat (K51A) [56,57,58], which were generously gifted by Dr. Zachary Klase, were carried out as previously described for the initial FDA-approved library screen. Then, the Bright Glo Luciferase Assay was carried out with luminescence being read using an integration time of 0.5 s on a Promega Glomax Multi Detection System.

### 2.4. Cell Viability Assay

Fifty thousand cells were plated per well in a 96-well plate and treated the next day with 1.0 or 10 μM compound or DMSO. Forty-eight hours later, CellTiter-Glo (Promega, Madison, WI, USA) was used to measure the viability following the manufacturer’s recommendations. CellTiter-Glo is a luminescent assay used to measure cell viability by ATP level. The reagent was added to the wells (1:1 reagent:media) and incubated at room temperature for 10 min protected from the light. The luminescence was detected using the GloMax-Multi Detection System (Promega, Madison, WI, USA).

### 2.5. FDA-Approved Drugs and Other Chemicals

The 420 FDA-approved drugs came as a limited drug library from Selleckchem (Cat. No. L1300; Houston, TX, USA). Additional febuxostat (Cat. No. S1547), eltrombopag (Cat. No. S2229), resveratrol (Cat. No. S1396), SAHA (Cat. No. S1047), mycophenolic acid (Cat. No. S2487), and flavopiridol (Cat. No. S1230) were also purchased from Selleckchem and resuspended in DMSO or water as required. The three-drug antiretroviral therapy (ART) cocktail of lamivudine/emtricitabine, tenofovir and indinavir were obtained through the NIH AIDS Reagent Program, Division of AIDS, NIAID, NIH.

### 2.6. Protein Extracts and Immunoblotting

Cells were collected, washed twice with PBS and pelleted. Cells were lysed in a buffer containing 50 mM Tris–HCl pH 7.5, 120 mM NaCl, 5 mM ethylenediaminetetraactic acid (EDTA), 0.5% NP-40, 50 mM NaF, 0.2 mM Na_3_VO_4_, 1 mM dithiothreitol (DTT) and one tablet of complete protease inhibitor cocktail per 50 mL. Lysis was performed on ice for 30 min with vortexing every 5 min and spun at 4 °C for 5 min at 14,000 rpm. Supernatants were retained and the protein concentration for each preparation was determined with a Bio-Rad protein assay kit (Bio-Rad Laboratories, Hercules, CA, USA). Cell extracts (25 µg/sample) were resolved by SDS PAGE on a 4–20% tris–glycine gel (Invitrogen, Carlsbad, CA, USA). Proteins were transferred to polyvinylidenedifluoride microporous membranes overnight using a wet transfer tank system run at 80 mAmp. Membranes were blocked with Dulbecco’s phosphate-buffered saline (PBS) 0.1% Tween-20 + 3% BSA. Primary antibody against the specified protein was incubated with the membrane in a blocking solution overnight at 4 °C. Membranes were washed thrice with PBS + 0.1% Tween-20 and incubated with horseradish peroxidase (HRP)-conjugated secondary antibody for 2 h in blocking solution. The presence of secondary antibody was detected by SuperSignal West Dura Extended Duration Substrate (Pierce, Rockford, IL, USA). Luminescence was visualized on a Kodak 1D image station (Carestream Health, Rochester, NY, USA).

### 2.7. RNA Extraction, RT-PCR and qRT-PCR

For the mRNA analysis of the proviral transcripts, total RNA was isolated from cells using Trizol extraction (ThermoFisher Scientific, Grand Island, NY, USA) according to the manufacturer’s instructions. Five hundred nanograms of total RNA was used to generate cDNA with the iQ Supermix kit (Bio-Rad) using oligo-dT reverse primers. The full length and short abortive proviral transcripts were then quantified by qRT-PCR using envelope (ENV) and TAR responsive gene primers, respectively, that have been previously described [59]. The qRT-PCR assays were performed using the C1000 Thermo Cycler with the CFX96 Real-Time System and iTaq Universal SYBR Green Supermix (Bio-Rad). Cycling conditions were as follows: 1 cycle at 50 °C for 2 min, 1 cycle at 95 °C for 3 min, and 44 cycles at 95 °C for 15 s and 60 °C for 40 s. The absolute quantification was calculated based on the threshold cycle (Ct) relative to the standard curve and then the copy number was normalized based on the amount of RNA input into the RT reaction.

### 2.8. Jurkat E4 GFP Analysis by Plate Reader and Flow Cytometry

The Jurkat E4 clone, which harbors a mutated latent HIV-1 genome with the Gag gene deleted and the Nef gene replaced by d2EGFP [46], was treated with various concentrations of the top HIV-1 activators (0.5, 1.0 and 2.0 μM) and SAHA was used as a positive control. The GFP activation was then measured by either flow cytometry or plate reader at 24, 48 and 72 h post-treatment. For measuring GFP activation on a plate reader, the Blue optical kit (excitation of 490 nm, and emission detection of between 510 and 570 nm) was used with the Promega Glomax Multi Detection System to measure culture fluorescence. Alternate multi-dose regimens were also tested whereby either one or two additional doses of experimental activator or SAHA were added every 24 h after the first treatment.

### 2.9. ChIP Assay

Log phase growing cells, treated with flavopiridol, mycophenolic acid, eltrombopag or DMSO, were harvested and their DNA were cross-linked and processed using the Imprint Chromatin Immunoprecipitation Kit (Sigma, St. Louis, MO, USA). Samples were then sonicated and mono-disomes were used for immunoprecipitation. Antibodies were added, and the samples were allowed to rotate overnight at 4 °C. A 30% (*v*/*v*) protein A-Sepharose/protein G-Sepharose mix was added and allowed to rotate for 2 h at 4 °C. The samples were washed twice with IP Wash Buffer (Sigma, St. Louis, MO, USA) prior to the addition of Proteinase K (800 U/mL). After 15 min incubation at 65 °C, reversing solution (Sigma, St. Louis, MO, USA) was added and the samples incubated at 65 °C for 90 min. DNA was purified and real-time qPCR was performed using the NF-κB site 1 forward primer and TAR +59R Reverse Primer.

## 3. Results

### 3.1. Identification of Lead HIV-1 Transcriptional Activators from TZM-bl Screen of FDA-Approved Drugs

**Figure 1:**
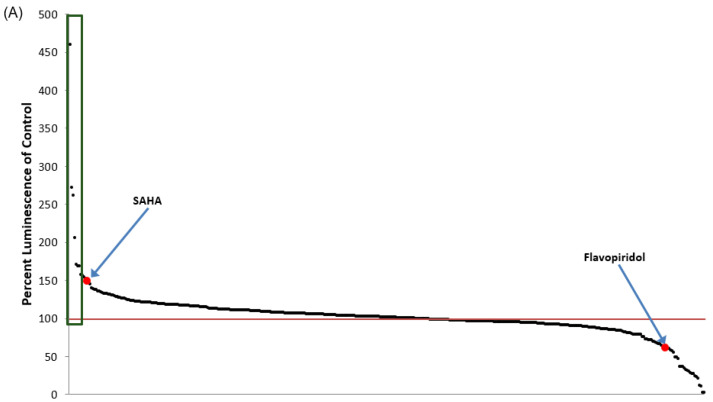

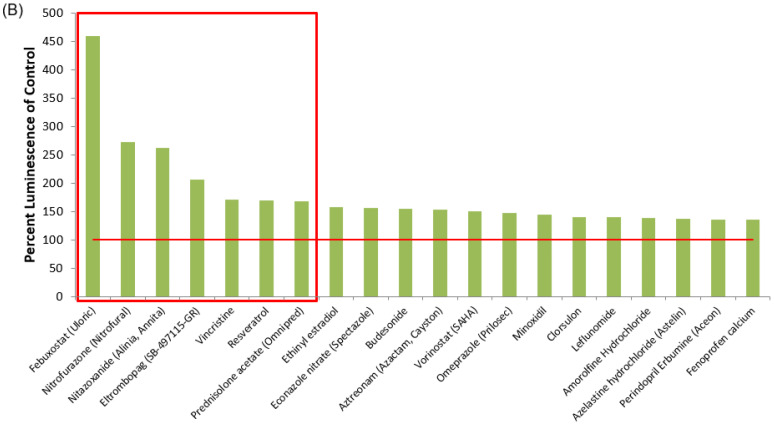
Initial identification of the activators of TZM-bl drug screen. (**A**) Ranked plot of all 420 FDA-approved drugs screened against pcTat-activated TZM-bl reporter cells. The average luminescence reading of the DMSO controls was set to 100% (red line) and all experimental readings were normalized to the DMSO control. Control transcriptional activator SAHA and control transcriptional inhibitor flavopiridol are indicated and the top seven activators are within the green box. (**B**) Ranked plotting and identification of the top 20 activators of the pcTat-TZM-bl reporter assay. The top 7 activators are indicated in the red box.

For testing the activation and inhibition of HIV-1 proviral transcription by the panel of 420 FDA-approved drugs, the TZM-bl cell line was utilized. TZM-bl is a HeLa-based cell line that harbors an integrated HIV-1 LTR-driven luciferase reporter gene [44]. Prior to screening the FDA-approved drugs, the optimization of the fast-forward transfection conditions of pc-Tat into the TZM-bl cell line was carried out to ensure the reporter assay gave a Z-factor greater than 0.5. The optimized pc-Tat transfection of TZM-bl cells described in Materials and Methods yielded a Z-factor of 0.85 indicating its suitability for the drug screen. For the initial screen, TZM-bl cells were plated and transfected with pc-Tat, then incubated overnight before replacing the media and treating the transfected cells with the 420 drugs at a final concentration of 1 μM. Luciferase activity was then measured 48 h post-treatment. Additionally, each plate contained at least four 0.01% DMSO-treated controls and four transcriptional inhibitor control wells treated with the P-TEFb inhibitor flavopiridol. For flavopiridol-treated controls in the TZM-bl screen, a concentration of 100 nM was used as the lethal dose, 50% (LD_50_) and half maximal inhibitory concentration (IC_50_) of flavopiridol were found to be 225 nM and 9.5 nM, respectively, in a previous study of a related HeLa cell line [60]. Therefore, 100 nM is at half the LD_50_ and 10-fold greater than the IC_50_, thereby minimizing toxicity while allowing for robust transcriptional inhibition. Figure 1A shows a ranked plot of the entire 420 FDA-approved drugs, as well as a detailed graph of the top 20 activators of pc-Tat-transfected TZM-bl (Figure 1B), ranked in order from greatest to least luciferase activation. A ranked listing of the top 20 activators of the TZM-bl drug screen is also given in Table 1 along with a brief description of the putative mechanism of action and/or approved indication for each compound. The percent luminescence obtained from the addition of the experimental compounds is normalized to the DMSO controls with the DMSO controls being set to 100%. Collectively, the TZM-bl reporter cell line screen identified the most efficacious HIV-1 transcriptional activators to be further validated and subsequently tested in HIV-1-infected cells.

### 3.2. Lead HIV-1 Activator pc-Luciferase and Toxicity Screens

**Figure 2:**
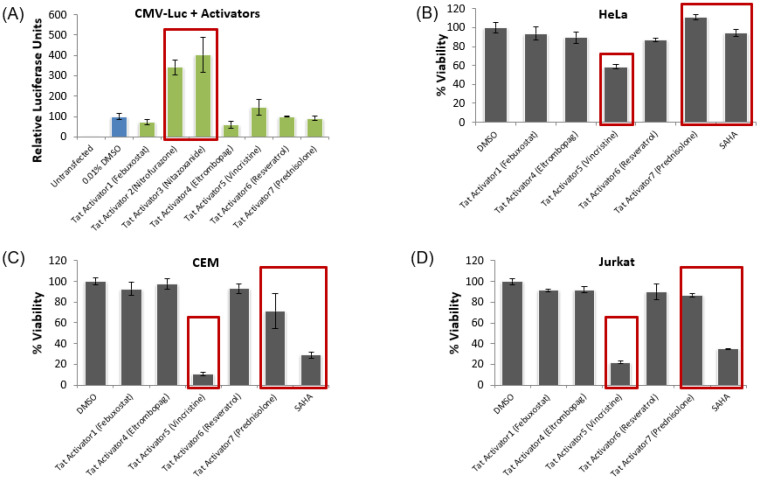
Cytomegalovirus (CMV)-luciferase activation and toxicity screens. (**A**) The top seven activators identified in the initial TZM-bl screen were tested in pc-Luc-transfected HeLa cells to determine the activation of the CMV promoter. (**B**–**D**) The five lead activators that did not activate the CMV promoter were tested for toxicity in the HeLa (**B**), CEM (**C**), and Jurkat (**D**) cell lines. All experimental conditions were conducted in biological triplicate and error bars indicate ±1 SD. Conditions marked by red boxes indicate *p* values < 0.05 when compared to DMSO treatment.

After identifying the top potential activators of HIV-1 transcription, the validation of the provirus-specific transcriptional activation was conducted. Specifically, we screened the top seven activators of the TZM-bl reporter system against HeLa cells transfected with a pc-Luciferase (pc-Luc) plasmid. This validation experiment was to identify any of the top seven activators that functioned by the upregulation of the pc-Tat vector used in the initial TZM-bl screen. In this validation experiment, the HeLa cell line was used as it is the parental cell line of TZM-bl and the pc-Luc plasmid has the same cytomegalovirus (CMV) promoter used to drive the expression of the Tat gene in the pc-Tat plasmid. Therefore, we can identify any drugs that increase CMV promoter activity, and thereby, would have increased levels of Tat in the original TZM-bl drug screen giving a false positive indication of enhancing proviral transcription. In this validation study, transfection conditions were identical to the original TZM-bl screening of the FDA-approved drugs and all drugs were dosed at 1 μM one day post-transfection, followed by luciferase activity quantification two days post-treatment. The data show that two of the activators of the TZM-bl reporter system, specifically nitrofurazone and nitazoxanide, activated the pc-Luc vector in a statistically significant manner (Figure 2A). Therefore, this validation experiment demonstrates that a couple of the initial hits in our screen of HIV-1 transcriptional activators were due to the upregulation of the CMV promoter of the pc-Tat plasmid used in the initial TZM-bl screen and these drugs were removed from further consideration. Furthermore, it shows that the remaining drugs that did not activate the CMV promoter also do not likely activate the HIV-1 provirus through NF-κB activation as the CMV promoter contains NF-κB enhancer elements [61,62,63].

We then conducted toxicity screens with the five remaining lead HIV-1 proviral activators that did not definitively upregulate the pc-Luc reporter. The toxicity screens were again conducted on HeLa, as well as Jurkat and CEM cell lines, in order to eliminate any of the remaining drug targets that are either toxic or affect the cell cycle of the initial screening cell line or uninfected T cells more relevant to patients. Again, all drugs were dosed at 1 μM one day after plating the cells, and then the cell viability was quantified using the Cell Titer Glo assay two days post-treatment. Based on the results, we found that one of the Tat activators, vincristine, was toxic to all three cell lines (Figure 2B–D). Additionally, prednisolone and the control activator SAHA decreased the viability in both of the T cell lines (Figure 2C,D). In sum, our results show that some of the remaining HIV-1 transcriptional activators are toxic to relevant cell lines of interest and these drugs were not further tested. Furthermore, our lead HIV-1 activators that did not activate the CMV promoter and were not cytotoxic were febuxostat, eltrombopag, and resveratrol. These three drugs have primary mechanisms of action that differ significantly. Specifically, febuxostat is a non-purine xanthine oxidase (XO) inhibitor [64,65], eltrombopag is a thrombopoietin receptor (TpoR) agonist [66], and resveratrol is a potent antioxidant isolated from the *V. vinifera* grape [67]. Of these three compounds, only resveratrol has been previously identified as a potential HIV LRA [68], therefore these experiments are the first to our knowledge that demonstrate that febuxostat and eltrombopag reverse HIV latency. Based on their performance in our initial round of assays, these three compounds were carried forward for further experimentation in additional cell line models of HIV-1 latency.

### 3.3. Testing Lead Transcriptional Activators in Latently HIV-1-Infected Cell Lines

Based on our preliminary results, we then tested the activation of viral replication in the latently HIV-1-infected ACH2 and OM10.1 cell lines using febuxostat, eltrombopag, and resveratrol. The selection of the ACH2 and OM10.1 cell lines in these transcriptional activation experiments allowed for testing in both an HIV-infected T cell and myeloid-derived cell line, respectively, thereby covering the most relevant cell types in the infected patient population. Additionally, a known activator of transcription, SAHA [69,70], was included as a positive control, and DMSO treatment served as a negative control for statistical comparison in this study. Moreover, the treatment of each of the cell lines was carried out both in the absence of ART pretreatment or after 11 days of ART treatment (lamivudine/emtricitabine, tenofovir, and indinavir, each at 10 µM, dosed every other day). For all cell line treatments, the experimental and control compounds were added to the test cell lines at day 0 and the cell pellet and supernatants were collected 48 h later. Isolated RNA from the cell pellets was then used to measure the full-length viral transcripts (both unspliced and singly spliced) by qRT-PCR using primers targeting the HIV-1 envelope (ENV) region and a copy number was normalized by the concentration of RNA input into the RT reaction. All experimental and positive control samples were compared against the DMSO controls to identify the statistically significant upregulation of viral transcription using the Student’s *t*-test.

**Figure 3:**
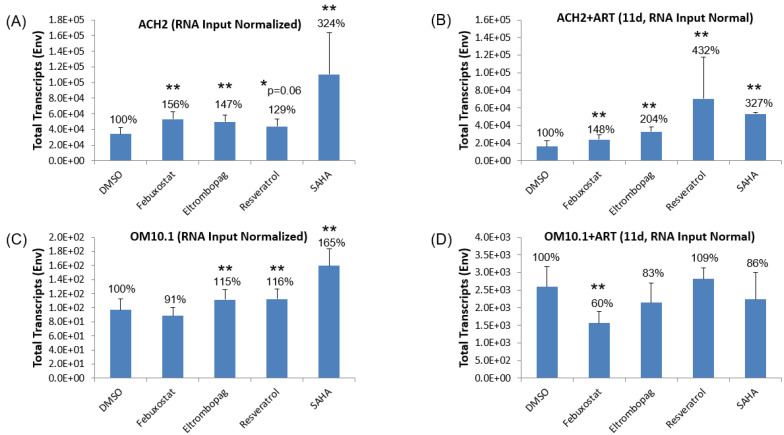
Transcriptional activation of latent ACH2 and OM10.1 cell lines. The three remaining lead activators were used to treat the latently HIV-1-infected ACH2 T cell line in the absence of antiretroviral therapy (ART) pre-treatment (**A**), or after 11 days of ART treatment (**B**). Similarly, the latently HIV-1-infected OM10.1 promyelocytic cell line was also treated with the lead activators in the absence (**C**) or presence of ART treatment (**D**). RNA was isolated from cellular pellets by Trizol extraction for each sample and the intracellular viral transcripts were quantified by RT-qPCR using primers for the envelope (ENV) gene. Total transcripts for each experiment were normalized to the RNA concentration input into each RT reaction. All experimental conditions were conducted in biological triplicate and error bars indicate ± 1 SD. ** indicates *p* value < 0.05 when compared to the DMSO treatment.

The analysis of the ENV transcript levels in ACH2 cells, shown in Figure 3A,B, demonstrates a statistically significant 2-fold activation by the positive control activator, SAHA, regardless of ART pretreatment, although there is significant variability in the cultures not treated with ART. Similarly, febuxostat showed a similar statistically significant increase in ENV transcripts regardless of ART pretreatment (56% versus 48%, no ART and ART pretreatment, respectively). Interestingly, the other two experimental compounds showed statistically significant differences in activation based on ART pretreatment. Specifically, eltrombopag showed a 47% increase in full-length proviral transcription untreated ACH2 samples but a doubling of viral transcripts after ART pretreatment (*p*-value = 0.009). Moreover, resveratrol has an even greater increase in activation after 11-day ART going from a 29% increase without ART to a greater than 3-fold increase after ART pretreatment (*p*-value = 0.05). These data suggest that two of the lead compounds, eltrombopag and resveratrol, may have drug–drug interactions with the 3-drug ART cocktail used that enhanced their ability to activate proviral transcription.

In contrast to the treatment of the latent ACH2 T cell line, activation with both the experimental and SAHA control was much less robust in the latent promyelocytic cell line OM10.1 (Figure 3C,D). Specifically, eltrombopag and resveratrol both demonstrated a less robust but statistically significant 15% increase in HIV-1 transcription in OM10.1 without ART pretreatment, while SAHA gave a 65% increase in ENV transcripts. After ART pretreatment, however, no drug, including SAHA, increased OM10.1 proviral transcription above DMSO control levels, and the experimental compound febuxostat actually reduced ENV copies. The reduced efficacy of all test compounds in OM10.1 versus ACH2 latent cell lines indicates potential differences in the transcriptional machinery in lymphoid- versus myeloid-derived cell lines. Differences in activation by LRAs in HIV-1-infected primary cells of different lineages has already been described, where HDACis that activate the latent provirus in quiescent CD4+ T cells actually suppress viral production in macrophages due to the activation of autophagy [37].

### 3.4. Testing Jurkat E4 Latent T Cell Model with Single and Multi-Dose Regimens of Lead HIV-1 Transcriptional Activators

**Figure 4:**
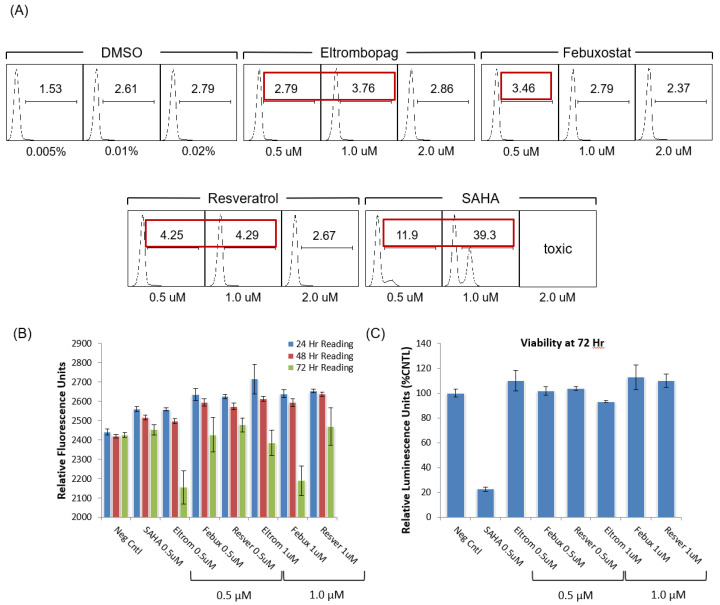
Single-dose of activators with the Jurkat E4 latent reporter cell line. The three remaining activators were tested against the latent reporter cell line Jurkat E4. (**A**) Flow cytometry data indicate GFP activation by all three activators at 48 h post-treatment at either 0.5 or 1.0 µM concentrations. (**B**) Similarly, testing GFP activation using a plate reader shows activation at 24 and 48 h post-treatment with both 0.5 and 1.0 µM concentrations but a rapid loss of activation by 72 h. (**C**) Lastly, the viability of the Jurkat E4 cell line was not negatively impacted by the single-dose administration of either 0.5 or 1.0 µM of the experimental activators, although 0.5 µM of SAHA was highly toxic.

An alternate method for examining HIV-1 transcriptional activation was also tested with our top activators. Here, the Jurkat E4 clone, which harbors a mutated latent HIV-1 genome with the Gag gene deleted and the Nef gene replaced by d2EGFP [46], was treated with various concentrations of the top Tat activators (0.5, 1.0 and 2.0 μM) and SAHA. The GFP activation was then measured using flow cytometry at 48 h post-treatment. The flow cytometry readings at 48 h post-treatment showed activation by all three experimental drugs at either the 0.5 or 1.0 μM concentrations, but not at the higher 2 µM levels (Figure 4A). In order to assess the time-dependent activation by the three experimental activators, the 0.5 and 1.0 µM treatments were repeated in the 96-well plate format, using a plate reader to measure GFP activation at 24, 48 and 72 h post-treatment. Here, the time-course measurements using the plate reader showed the highest fluorescent signal for each drug within 24 h of treatment with no fluorescence activation detected by 72 h post-treatment (Figure 4B).

**Figure 5:**
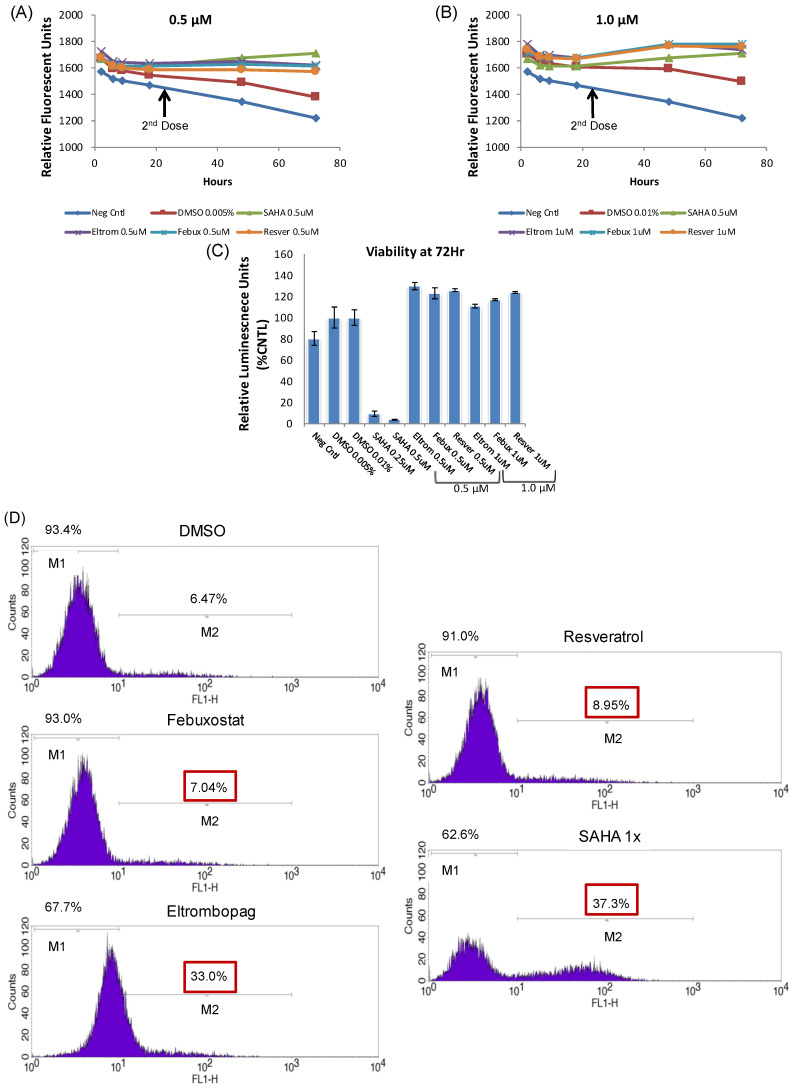
Multi-dose regimen of activators with the Jurkat E4 latent reporter cell line. (**A**,**B**) The addition of a second dose of each activator 24 h after the initial dose allowed for the sustained activation out to 72 h post-initial dosing (0.5 µM panel A, 1.0 µM panel (**B**). (**C**) Viability of the Jurkat E4 cell line was still not inhibited by the addition of a second dose of experimental activator, while multiple doses of SAHA were highly toxic. (**D**) Flow cytometry data indicate GFP activation by all three activators at 72 h after the initial treatment using three doses of 1.0 µM at 0, 24 and 48 h.

Based on the initial results from the single dose experiments, a multiple dose regiment was then tested with our experimental compounds, whereby the drug was added immediately after plating the Jurkat E4 cells and then a second dose was administered 24 h after the first treatment. Again, concentrations of 0.5 and 1.0 µM were used in the two-dose regimen and both concentrations allowed for an enhanced activation out to 72 h (Figure 5A,B). Furthermore, none of the three experimental compounds showed toxicity with either one or two doses, while a single dose of 0.5 μM SAHA-treated cells were only 23% of the negative control viability, and two 0.5 μM SAHA doses reduced viability to 4% (Figure 4C and Figure 5C). The SAHA toxicity was consistent with the toxicity previously observed in the uninfected CEM and Jurkat T cell lines (Figure 2C,D). Based on the initial results from the 96-well plate experiments, additional flow cytometry measurements were tested at 72 h using a multiple dose regimen and each showed differing levels of activation with the most significant fluorescence shift observed with eltrombopag (Figure 5D). While eltrombopag is comparable to SAHA in terms of the percentage of cells shifting into the positive gate, the mean fluorescent intensity of the GFP-positive cells is much higher in the SAHA-treated cells than the eltrombopag-treated cells. Overall, the use of a multi-dose treatment regimen for each of these three experimental activators is a viable treatment option due to the lack of toxicity and enhanced proviral activation was observed. Moreover, the final concentration of drug added after the final dose in these in vitro experiments is still lower than the plasma C_max_ observed in patients given a single dose of each of these drugs in clinical trials [71,72,73]. Additionally, those compounds demonstrating significant changes in the transcriptional activation of the ACH2, OM10.1, and Jurkat E4 cell lines were considered for subsequent experiments with infected primary cell cultures.

### 3.5. Identification of Lead HIV-1 Transcriptional Inhibitors from TZM-bl Screen of FDA-Approved Drugs

**Figure 6:**
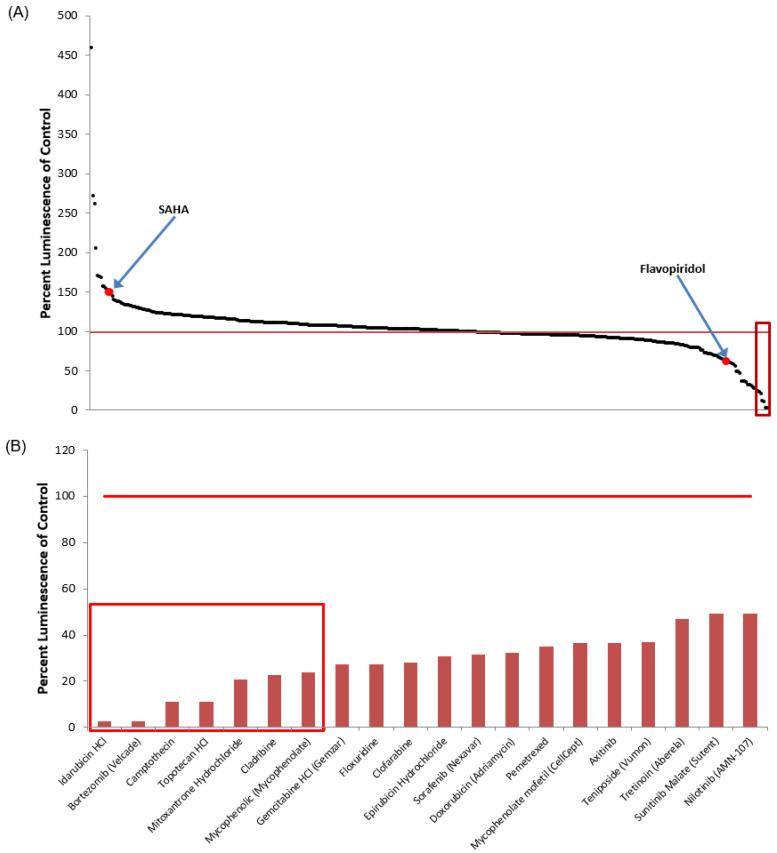
Initial identification of inhibitors of TZM-bl drug screen. (**A**) Ranked plot of all 420 FDA-approved drugs screened against pcTat-activated TZM-bl reporter cells. The average luminescence reading of the DMSO controls was set to 100% (red line) and all experimental readings were normalized to the DMSO control. Control transcriptional activator SAHA and control transcriptional inhibitor flavopiridol are indicated and the top seven inhibitors are within the red box. (**B**) The ranked plotting and identification of the top 20 inhibitors of the pcTat-TZM-bl reporter assay. The top 7 inhibitors are indicated in the red box.

From the initial screen of the library of 420 FDA-approved drugs on pc-Tat transfected TZM-bl, we then turned our focus on those compounds that inhibited HIV-1 transcription. Figure 6A shows the same ranked plot of the entire 420 FDA-approved drugs as Figure 1A with the top inhibitors highlighted. A detailed graph of the top 20 inhibitors of pc-Tat transfected TZM-bl is then shown in Figure 6B, ranked in order from greatest to least luciferase inhibition. A ranked listing of the top 20 inhibitors of the TZM-bl drug screen is also given in Table 2 along with a brief description of the putative mechanism of action and/or approved indication for each compound. Interestingly, four of the top seven inhibitors were topoisomerase inhibitors, a class of drugs that has long been identified as HIV replication inhibitors [74,75]. In Figure 6 and Table 2, the percent luminescence obtained from the addition of the experiment compounds is normalized to the DMSO controls with the DMSO controls being set to 100%. Collectively, the TZM-bl reporter cell line screen identified the most efficacious HIV-1 transcriptional inhibitors to be further validated and subsequently tested in infected cells.

### 3.6. Lead HIV-1 Inhibitor pc-Luciferase and Toxicity Screens

**Figure 7:**
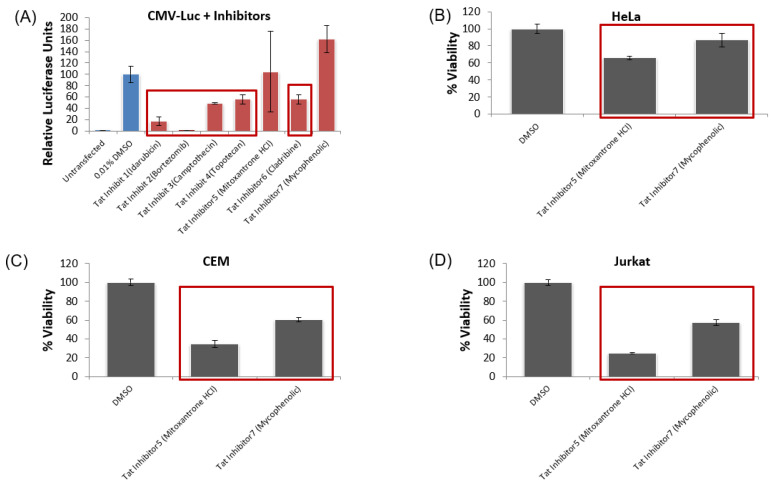
CMV-luciferase inhibition and toxicity screens. (**A**) The top seven inhibitors identified in the initial TZM-bl screen were tested in pc-Luc-transfected HeLa cells to determine the inhibition of the CMV promoter. (**B**–**D**) The two lead inhibitors that did not inhibit the CMV promoter were tested for toxicity in the HeLa (**B**), CEM (**C**), and Jurkat (**D**) cell lines. All experimental conditions were conducted in biological triplicate and error bars indicate ±1 SD. Conditions marked by red boxes indicate *p* values < 0.05 when compared to DMSO treatment.

Similar to the HIV-1 transcriptional activator leads, the validation of provirus-specific transcriptional inhibition of the top potential inhibitors of HIV-1 proviral transcription was conducted. Specifically, we screened the top seven inhibitors of the TZM-Bl reporter system against HeLa cells transfected with a pc-Luc plasmid. This validation experiment was to identify any of the top seven inhibitors that functioned by either the downregulation of the CMV promoter used by the pc-Tat plasmid in the initial FDA drug screen, cytotoxicity, or changes to cell cycle regulation. Again, all the transfection and drug treatment conditions were identical to the initial screen, whereby drugs were dosed at 1 μM one day post-transfection, and then the luciferase activity was quantified two days post-treatment. Of the lead inhibitors of pcTat-TZM-bl luciferase expression, five inhibited the expression of the CMV-Luc vector in a statistically significant manner, namely idarubicin HCl, bortezomib, camptothecin, topotecan HCl, and cladribine (Figure 7A). These validation experiments demonstrated that many of the initial hits in our screen Tat-mediated transcriptional inhibitors were likely due to either the altered regulation of the CMV promoter found on the pc-Tat plasmid cytotoxicity, or changes in the cell cycle.

We then conducted toxicity screens with the remaining HIV-1 inhibitors that did not definitively downregulate the pc-Luc reporter. These toxicity screens were again conducted on HeLa, as well as Jurkat and CEM cell lines, in order to eliminate any of the remaining drug targets that were either toxic or affected the cell cycle of the initial screening cell line or the target uninfected T cells in subsequent studies. Again, all the drugs were dosed at 1 μM one day after plating the cells, and then the cell viability was quantified using the Cell Titer Glo assay two days post-treatment. Based on the results, we found that both of the remaining Tat inhibitors, mitoxantrone HCl and mycophenolate, reduced the viable cell density of all three cells lines in a statistically significant manner, although mycophenolate only reduced the viability of HeLa cells by 13% (Figure 7B–D). Since previous toxicity screening utilizing mycophenolate has shown that it does not negatively impact primary PBMC viability up to a dose of 20 µM [76], we decided to continue testing it in subsequent experimental Tat inhibitor studies. In sum, our pc-Luc and toxicity screen results show that all of the top seven HIV-1 inhibitors from the initial FDA drug screen either inhibit the CMV promoter or are toxic to the relevant cell lines tested. Mycophenolate, an inhibitor of inosine-5′-monophosphate dehydrogenase (IMPD) [77], has previously been shown to be non-toxic to patient-relevant PBMC cultures and has previously been identified as an inhibitor of HIV replication [78,79]. Since it only had minimal toxicity in HeLa cells, it was moved forward for additional testing.

### 3.7. Testing Lead Transcriptional Inhibitors in Chronically HIV-1-Infected Cell Lines

Based on our preliminary results, we tested the inhibition of proviral transcription in the chronically HIV-1-infected J1.1 and OM10.1 cell lines with mycophenolate. Again, both the J1.1 and OM10.1 cell lines were examined as they are T cell and myeloid in origin, respectively, representing two important host cell types infected by HIV-1. Furthermore, the J1.1 cell line is a highly productive infected cell line that generates large quantities of wild-type virus and therefore, is a strong model to test transcriptional inhibitors. Additionally, a known inhibitor of transcription, flavopiridol (100 nM), was included as a positive control, and DMSO treatment served as a negative control for statistical comparison in this study. Moreover, drug treatment was carried out in both cell lines in either the presence or absence of 48 h of 10 nM PMA pretreatment to stimulate viral production. The enhanced viral replication by PMA activation thereby represents a worst case scenario on which we can test our lead transcriptional inhibitor, mycophenolate. As with the transcriptional activator cell line experiments, RNA isolated from cell pellets 48 h after treatment was used to measure the viral transcripts by RT-qPCR using primers amplifying HIV-1 ENV with a copy number normalized by the concentration of RNA input into the RT reaction. All experimental and positive control samples were compared against the DMSO controls to identify the statistically significant downregulation of viral transcription using the Student’s *t*-test.

**Figure 8:**
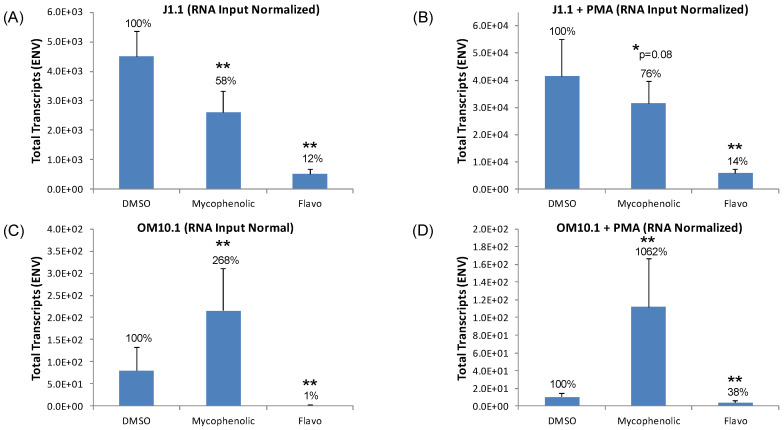
Transcriptional inhibition of chronically infected J1.1 and OM10.1 cell lines. The remaining lead inhibitor, mycophenolate, and control inhibitor flavopiridol, were used to treat the chronically HIV-1-infected J1.1 T cell line and intracellular ENV copies were quantified by RT-qPCR and normalized to the RNA input into the RT reaction. The J1.1 drug treatment occurred either in the absence of PMA activation (**A**), or 48 h post-PMA treatment (**B**). Similarly, the HIV-1-infected OM10.1 promyelocytic cell line was also treated with mycophenolate or flavopiridol in the absence (**C**) or presence of PMA pre-treatment (**D**). All experimental conditions were conducted in biological triplicate and error bars indicate ± 1 SD. ** indicates *p* value < 0.05 when compared to DMSO treatment.

Analysis of the ENV transcript levels in J1.1 cells, shown in Figure 8A,B, demonstrates a statistically significant 86 to 88% reduction of ENV copies by the positive control inhibitor, flavopiridol, regardless of PMA stimulation. Mycophenolate also elicited a 42% decrease in ENV transcripts in the non-PMA-treated J1.1 cells, but its level of inhibition was reduced to only 24% after PMA activation. Similar to the J1.1 inhibitor experiments, we observed potent reductions in ENV copies by flavopiridol in both PMA-treated and untreated OM10.1 cells (Figure 8C,D). Conversely, the mycophenolate treatment actually caused statistically significant 2- and 10-fold increases in ENV copies in untreated and PMA-pretreated OM10.1cells, respectively (Figure 8C,D). The opposite effect on ENV transcript levels by mycophenolate on J1.1 and OM10.1 cell lines again points to the potential differences in the transcriptional machinery in lymphoid- versus myeloid-derived cells. These findings also stand in contrast to a previous study that showed the inhibition of HIV replication in monocyte-derived macrophages when mycophenolate was added to abacavir treatment [78]. Since mycophenolate did demonstrate a statistically significant reduction in viral mRNA with the J1.1 cells we then carried it forward into subsequent infected primary culture experiments.

### 3.8. Eltrombopag and Mycophenolate Treatment of Infected T Cells and Macrophages

After testing the most potent modulators of HIV-1 transcription on relevant reporter and infected cell lines, we selected eltrombopag and mycophenolate as our lead activator and inhibitor, respectively, to test in the single-cycle infections of primary cultures. Eltrombopag showed the highest level of proviral transcriptional activation based on the combined qRT-PCR results from the various ACH2 and OM10.1 experimental conditions and the Jurkat E4 experiments. To the best of our knowledge, it is also a newly identified LRA, therefore, we proceeded to test it on the infected primary cells. Similarly, since qRT-qPCR results showed mycophenolate-suppressing J1.1 proviral transcription in various experimental conditions, it was used here, although it also activated OM10.1 transcription. To examine the effects of eltrombopag and mycophenolate on proviral transcription, dual tropic virus (89.6) was used to infect isolated T cells and monocyte-derived macrophages (MDMs). The infected cultures were then maintained in culture for ten days in the presence of ART, specifically lamivudine/emtricitabine, tenofovir and indinavir, each at 10 µM, dosed every other day. The experimental compounds were then administered at 1 µM after culturing the infected cells with ART and then cell pellets and supernatants were collected 72 h post-treatment. Again, isolated RNA from the cell pellets was used to measure viral transcripts by RT-qPCR and DMSO treatment was used as a negative control for both infected T cells and MDMs. Here, primers to both the ENV and transactivator response element (TAR) regions were used to determine the viral transcript levels intracellularly and copy numbers were normalized by the concentration of RNA input into the RT reaction. Here, the ENV copies represent the full-length transcripts (both unspliced and singly spliced) and the number of TAR copies minus the number of ENV copies indicates short abortive proviral transcripts. The measurement of TAR transcripts was utilized here as a first step in elucidating the molecular mechanism by which the experimental compounds may work. This is possible as the enhanced ratio of TAR to ENV shows a high level of Pol II recruitment without subsequent successful transcriptional elongation. Our lab has previously demonstrated that inhibitors of P-TEFb block phosphorylation of the CTD of Pol II, subsequently leading to the enhanced production of TAR RNA. Therefore, if higher TAR levels are observed in relation to ENV, it may indicate the inhibition of Pol II elongation. Alternately, proportional decreases or increases in TAR and ENV may indicate the inhibition or enhancement of Pol II recruitment to the viral promoter without an alteration of the transcriptional elongation machinery.

**Figure 9:**
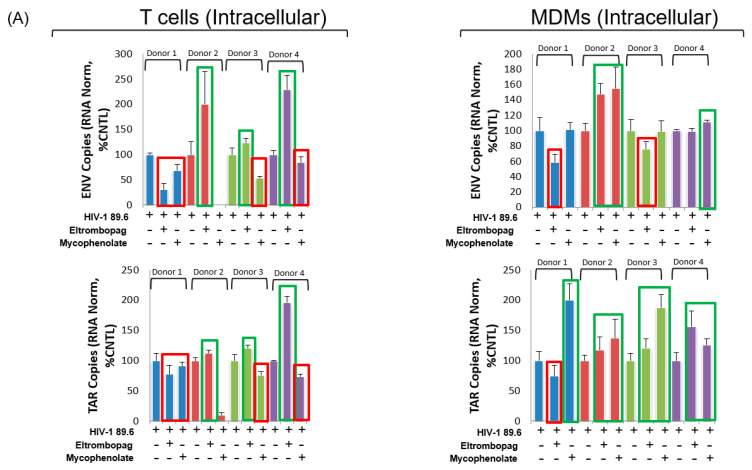

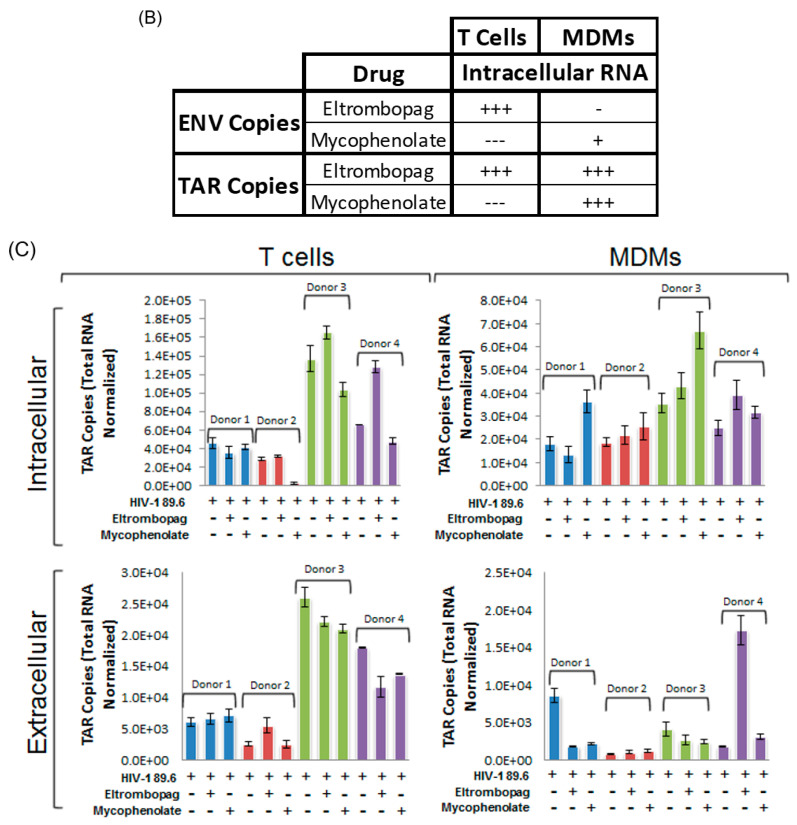
Transcriptional activation or inhibition of the infected primary T cells or monocyte-derived macrophages (MDMs) with lead modulators. T cells and MDMs were isolated from fresh peripheral blood mononuclear cells (PBMCs) from four donors, infected with 89.6 dual tropic HIV-1, then maintained on ART for 10 days. After ART treatment, both T cells ((**A**), **left panels**) and MDMs ((**A**), **right panels**) were either untreated, dosed with 1 µM of eltrombopag or 1 µM of mycophenolate. Copies of ENV and transactivating response (TAR) (upper and lower panels, respectively) were then measured from the cell pellets 72 h after treatment and normalized to the untreated control infections for each donor. The eltrombopag- and mycophenolate-treated T cells ((**A**), **left panels**) showed the activation or inhibition, respectively, of the ENV in 3 of 4 infected donors with concurrent and similar relative increases or decreases in TAR. The eltrombopag- and mycophenolate-treated MDMs ((**A**), **right panels**) did not show consistent activation or inhibition of infected MDMs, although TAR levels were enhanced in all drug-treated cells except the eltrombopag-treated donor 1. The green and red boxes highlight increases and decreases above the untreated controls, respectively, of ENV and TAR copies. (**B**) An overview of the findings for the intracellular ENV and TAR copies summarized in tabular form. Here, each “+” indicates an infected donor with a greater than 10% increase in ENV or TAR transcripts, while each “−” indicates an infected donor with a greater than 10% decrease in ENV or TAR transcripts. (**C**) Additionally, the number of TAR copies was determined both intracellularly (upper panels) and from the culture supernatant (lower panels) of both the T cells (left panels) and MDMs (right panels).

Our results testing eltrombopag on infected primary cells under ART pretreatment show that three of the four HIV-1-infected donor T cells had enhanced ENV transcript levels (Figure 9A, upper left panel). Concurrently, the levels of TAR transcripts increased at a comparable rate in these three donors, thereby indicating an overall activation of the proviral promoter without an alteration of the transcriptional elongation machinery (Figure 9A, lower left panel). Similarly, in all three HIV-1-infected donor T cells from which RNA was purified (no RNA purified from donor 2), mycophenolate suppressed viral transcription (Figure 9A, upper left panel). Again, ENV copies were reduced at a comparable level as TAR demonstrated a potential inhibition of the provirus promoter and not the inhibition of the transcriptional elongation machinery, such as P-TEFb (Figure 9A, lower left panel). Conversely, neither eltrombopag nor mycophenolate produced a meaningful or consistent activation or inhibition of ENV transcripts in the four HIV-1-infected donor MDMs (Figure 9A, upper right panel). This divergence in the two drugs’ activity between the two primary cell lineages again demonstrates potential differences in HIV-1 proviral transcription in infected T cells and MDMs. Interestingly, even though ENV levels did not change consistently in response to either eltrombopag and mycophenolate, an increase in TAR levels was observed with both drugs in infected MDMs (Figure 9A, lower right panel). This may indicate increased Pol II recruitment to the proviral promoter by both compounds but the subsequent inhibition of the transcriptional elongation in MDMs. Additionally, we were able to detect TAR in the extracellular supernatant of all T cell and MDM samples, with the T cell supernatants containing greater than a log higher concentration of the short abortive transcripts (Figure 9C). While neither the eltrombopag nor mycophenolate treatments had any significant effect on the number of copies in the culture supernatants, these results do confirm our previous findings of TAR being packaged into the exosomes of infected primary cells [59,80]. A summary of the findings from the primary cell experiments are found in tabular form in Figure 9B.

### 3.9. Elucidation of Mechanism of Action of Lead HIV-1 Activators and Inhibitors

**Figure 10:**
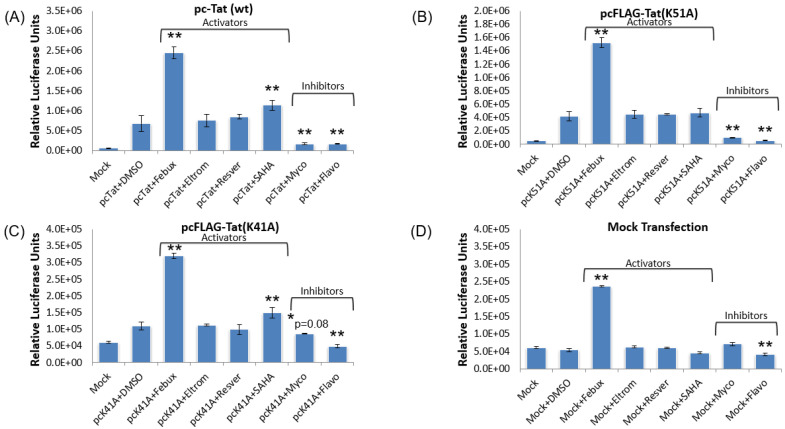
TZM-bl activation or inhibition with lead modulators using Tat mutants. The three lead HIV-1 activators well as the lead inhibitor were used to treat TZM-bl cells transfected with either wild-type pcFLAG-Tat (**A**), the K51A mutant of pcFLAG-Tat (**B**), the K41A mutant of pcFLAG-Tat (**C**), or mock transfections (**D**). Additionally, the control activator SAHA and the control inhibitor flavopiridol (100 nM) were tested in all four experimental settings. All drugs were dosed at 1 µM the day after transfection, and luciferase activity was measured 48 h after drug treatment. All experimental conditions were conducted in biological triplicate and error bars indicate ±1 SD. ** indicates *p* value < 0.05 when compared to the DMSO treatment of the same plasmid transfection.

After demonstrating the utility of eltrombopag and mycophenolate in HIV-1-infected primary T cells and MDMs, the mechanism(s) of action of the lead HIV-1 transcriptional modulators were then examined. Here, the TZM-bl reporter cell line used in the initial screen of the 420 FDA-approved drugs was again utilized to screen the lead activators and inhibitors efficacy with different functional mutants of the viral transcriptional transactivator Tat. The two Tat mutants utilized were the K51A and K41A amino acid residue mutants. The K51A mutant eliminates an important site of acetylation and methylation, and modestly reduces the transcriptional transactivation of the provirus [56,57,58]. On the other hand, the K41A mutant in the Tat activation domain strongly abrogates proviral transcriptional initiation and elongation by blocking the recruitment of transcription factors, such as P-TEFb, a requisite for the phosphorylation of the CTD of Pol II [52,53,54,55]. In this experiment, FLAG-tagged versions of the two Tat mutants were used to transfect TZM-bl cells. Additionally, mock transfections were also carried out to verify the necessity of Tat in the mechanism(s) of action of our lead transcriptional modulators. The day after transfection of the TZM-bl cells, treatment with 1 µM of the lead activators (febuxostat, eltrombopag, and resveratrol) and the lead inhibitor (mycophenolate) were carried out. Additionally, DMSO was used as a negative controls and SAHA (1 µM) and flavopiridol (100 nM) were used as the control activator and suppressor, respectively. In all four transfection conditions, febuxostat yielded a 1.9- to 3.5-fold increase in luciferase activity as compared to DMSO-treated controls transfected with the same plasmid or even in the absence of a plasmid (Figure 10A–D). Interestingly, the transfection condition where febuxostat treatment gave its lowest fold increase in luciferase activity was with the K41A Tat mutant, which may indicate that its activation of proviral transcription may be enhanced by the interaction with the Tat-mediated transcription (Figure 10C). On the other hand, febuxostat also showed robust activation in the total absence of Tat, demonstrating that its functionality is largely independent of the presence of Tat (Figure 10D). For the other two activators, eltrombopag and resveratrol, the highest activation for both was observed with wild-type Tat, while little to no effect was observed with either a Tat mutant or in mock transfections (Figure 10A–D). This indicates that both drugs likely work through enhancement of fully functioning proviral Tat-mediated transcription. Lastly, the transcriptional inhibitor mycophenolate generated comparable statistically significant luciferase activity reductions of greater than 75% in the Tat wild-type and K51A mutant transfections (Figure 10A,B) but little to no inhibition in the K41A and mock transfections (Figure 10C,D). This demonstrates that the mycophenolate inhibition of the provirus does appear to be dependent upon Tat-mediated transcription as basal levels of transcription were not significantly hindered in the K41A mutant and mock transfections.

**Figure 11:**
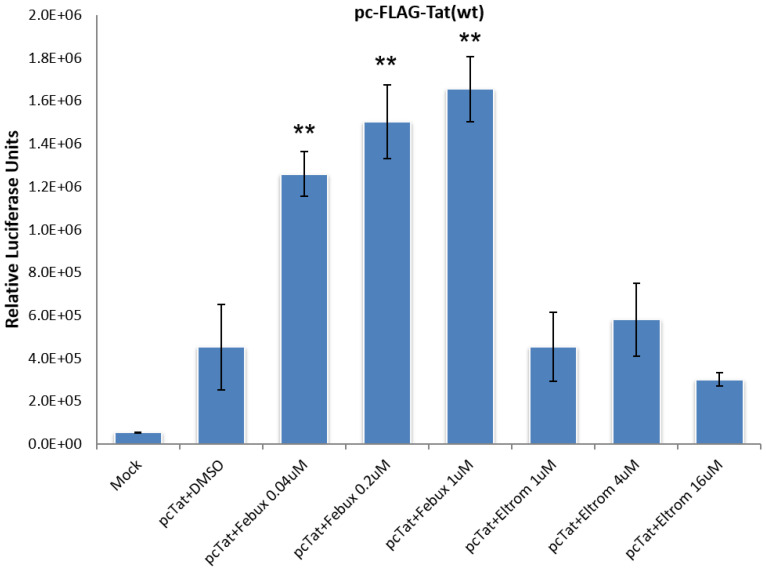
Dose-dependent activation of TZM-bl with the lead HIV-1 transcriptional activators. Two of the lead HIV-1 activators, febuxostat and eltrombopag, were added to Tat-transfected TZM-bl cells at three concentrations (0.04, 0.2, and 1 µM, or 1, 4, and 16 µM, respectively). Luciferase activity was measured 48 h after drug treatment and all experiments were run in biological triplicate. Mean relative luciferase units ± 1 SD graphed. ** indicates *p* value < 0.05 when compared to DMSO treatment of the same plasmid transfection.

We also wanted to demonstrate a dose-dependent response to our lead transcriptional activators. In order to test dose dependency, we carried out a three-point titration of two of the lead activators, febuxostat and eltrombopag (0.04, 0.2, and 1 µM, and 1, 4, and 16 µM, respectively), in the presence of Tat. The TZM-bl reporter cell line was again utilized and luciferase readings were taken 48 h post-treatment. A clear concentration was correlated with an increase in the activation of the TZM-bl cell line, observed across the three doses of febuxostat tested (Figure 11). Additionally, an increase in activation was observed from 1 to 4 µM of eltrombopag but was dose-limited at the highest 16 µM concentration (Figure 11). Collectively, these results indicate that the primary effect of both activators and inhibitors, with the exception of febuxostat, is likely mediate through Tat.

### 3.10. Levels of Transcription Factors and Occupancy on the HIV-1 Promoter

**Figure 12:**
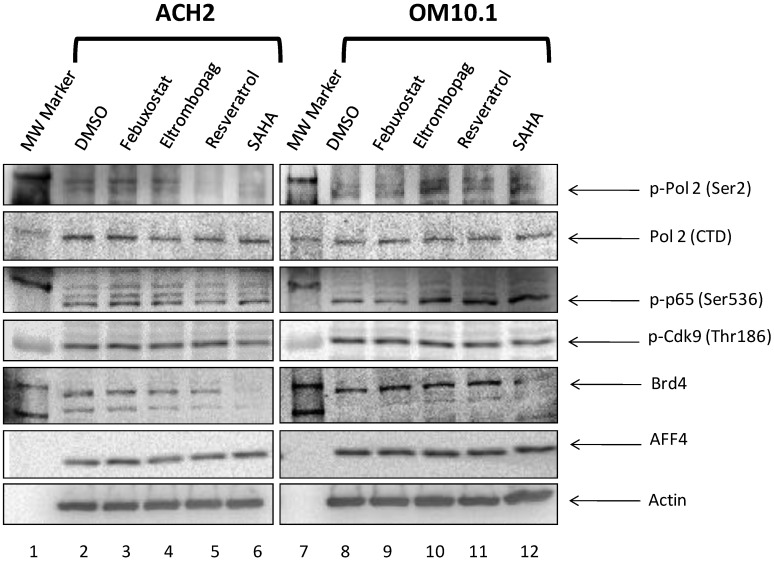
Western blot analysis of the lead HIV-1 transcriptional activators effects on key T cell and myeloid cell line host cell transcription factors. The three lead HIV-1 transcriptional activators, as well as the control activator SAHA, were used to treat either ACH2 or OM10.1 cell lines at 1 µM each. After 24 h of treatment, whole cell lysates were generated, run on Western blots and probed with antibodies binding to RNA polymerase 2 (phospho-Ser2), total RNA polymerase 2 (C-terminal domain (CTD)), p65 (phospho-Ser536), Cdk9 (phospho-Thr186), Brd4, and AFF4. Additionally, β-Actin was probed for as a loading control.

In order to identify the transcription factors involved with the transcriptional activators’ MOA, we carried out immunoblots of whole cell extracts from ACH2 and OM10.1 cells treated with 1 μM of our lead activators for 24 h. Here, we found that ACH2 cells treated with either febuxostat or eltrombopag had increased cellular levels of p-Pol II (Ser2) and p-p65 (Ser536) (Figure 12, left panel). With the treated OM10.1 cells, p-Pol II (Ser2) and p-p65 (Ser536) increased with eltrombopag and resveratrol treatment (Figure 12, right panel). For all experimental drug treatments on both ACH2 and OM10.1 cells, the total cellular levels of Pol II, p-Cdk9 (Thr186), Brd4, and AFF4 were unchanged. Interestingly, there was a dramatic downregulation of the observed Brd4 in the SAHA-treated ACH2 and OM10.1 cultures. The lower observed levels of Brd4 are likely due to the fact that SAHA increases nucleosome acetylation and therefore, the bromodomain of Brd4 is tightly bound to the acetylated histones and not released by our lysis conditions. Overall, these results indicate that increases in p-Pol II (Ser2) and p-p65 (Ser536) are consistent across ACH2 and OM10.1 cells treated with eltrombopag. Additionally, febuxostat increased p-Pol II (Ser2) and p-p65 (Ser536) in ACH2 cells, while resveratrol did the same in OM10.1, but neither drug produced the same outcome in both cell lines. This difference in signaling in response to drug treatment once again demonstrates the differences in transcription between T cells and cells of myeloid lineage.

**Figure 13:**
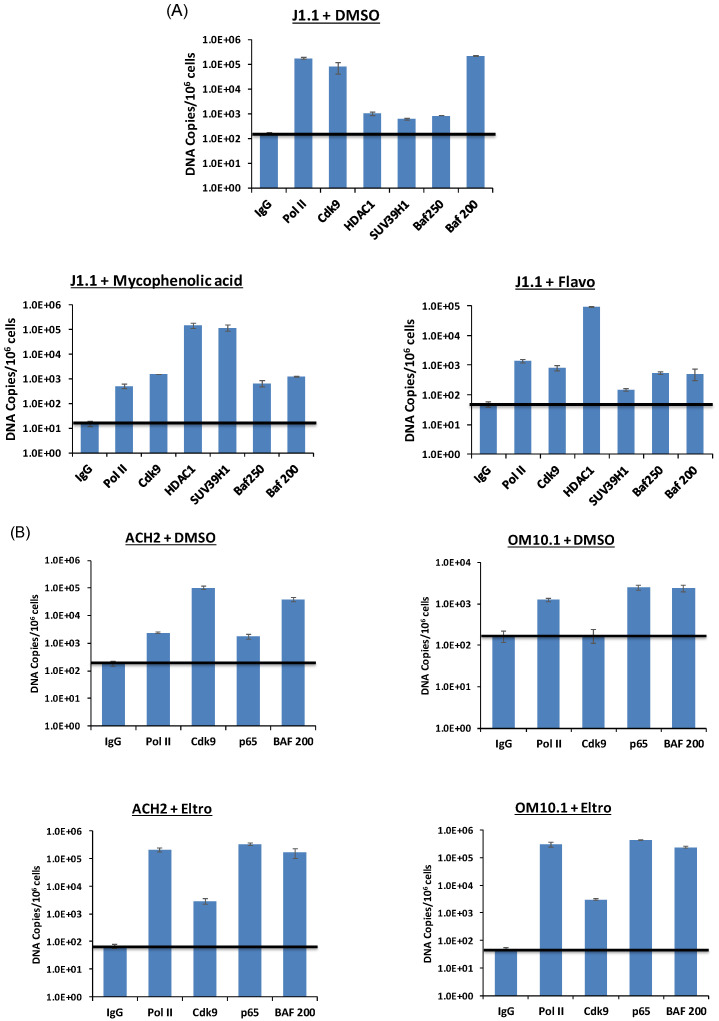
Transcription factor occupancy on the HIV-1 promoter following activation or suppression. (**A**) Infected J1.1 T-cells, which normally show a high level of transcription and virus production [81,82], were treated with either flavopiridol (100 nM) or mycophenolic acid. After 48 h, the samples were cross-linked and processed using a chromatin immunoprecipitation (ChIP) assay. Antibodies (~10 µg each) were used against Pol II, Cdk9, HDAC1, SUV39H1, Baf 200 and Baf 250. IgG was used as a control to define the background IP levels normally seen when performing ChIP assays. Primers for PCR assay were against the DNA sequence spanning the NF-κB element and TAR regions. (**B**) Both log phase growing ACH2 and OM10.1 cells that do not express much full-length viral RNA were treated with cART followed by eltrombopag treatment for 48 h prior to the ChIP assay using anti-Pol II, Cdk9, p65 and Baf200 antibodies.

Lastly, we questioned whether the promoter occupancy of certain transcription factors can be altered following either activator or inhibitor treatment. For the activator experiments, we used J1.1 T-cells, which are infected with a wild-type LAI strain of the virus and in which there is active transcription and viral shedding from these cells [81,82]. We treated these cells with either flavopiridol (100 nM; positive control) or our inhibitor mycophenolic acid. Samples were cross-linked 48 h post-treatment and used for chromatin immunoprecipitation (ChIP) using antibodies against Pol II, Cdk9, HDAC1, SUV39H1, and activator SWI/SIVF (PBAF; Baf 200) or suppressor SWI/SNF (BAF; Baf 250). The results in Figure 13A indicate that, as expected, Pol II, Cdk9 and Baf 200 were present on HIV-1 LTR in the absence of any activators (DMSO control). However, when treated with mycophenolic acid, levels of both HDAC1 and SUV39H1 increased indicating that histones (or other substrates) were regulated by deacetylation and methylation on the promoter. Interestingly, flavopiridol treatment increased only HDAC1 and not SUV39H1 levels, which indicates a different mechanism of inhibition as compared to mycophenolic acid.

We then treated ACH2 and OM10.1 cells with our activator eltrombopag and performed a similar ChIP assay. Data in Figure 13B indicate that PoI II and p65 levels increased (>2 logs each) on the HIV-1 promoter in ACH2 T-cells. A similar trend was also observed in OM10.1 myleoid cells where a dramatic increase in Pol II, Cdk9, p65 and Baf200 (~1.5–3.0 fold) factor occupancy was observed in these cells. Collectively, these data indicate that well known regulators of HIV-1 proviral transcription, such as Pol II, HDAC1, SUV39H1, p65 and members of SWI/SNF chromatin remodeling complex, are regulated by treatment with our lead activator and inhibitor.

## 4. Discussion

In the current manuscript, we screened 420 FDA-approved pharmaceuticals to identify those that either up or downregulated HIV-1 transcription. The major benefits of repurposing drugs approved for alternate indications is that the compounds have well established toxicity profiles in humans, approved manufacturing processes, and immediate commercial availability to the target patient populations. Here, we used a series of sequential assays to narrow down our list of lead compounds for eventual testing in infected primary cell cultures and alternate reporter assays. The screening assays used to funnel down our lead hits started with the initial HIV-1 LTR-luciferase reporter screen of the library of 420 FDA-approved drugs using the TZM-bl cell line transfected with viral transcriptional transactivator, Tat. A secondary pc-Luc transfection of HeLa cells was then used to eliminate those compounds affecting the CMV promoter of the pc-Tat plasmids used in the initial screen or altering cell cycle that would have resulted in false positive hits in the first screen. Lastly, the toxicity screening of three cell lines was conducted to remove any drugs that are toxic or otherwise affect the cell cycle of uninfected cell lines of interest. These sequential assays left us with a handful of lead candidates to move forward with into subsequent testing in relevant chronically infected cell lines, alternate reporter assays, and infected primary cells. The remaining lead compounds included three HIV-1 activators (febuxostat, eltrombopag, and resveratrol), as well as one HIV-1 inhibitor (mycophenolate). Of the lead modulators identified, to our knowledge, this is the first report of either febuxostat or eltrombopag demonstrating HIV transcriptional activation. It is also important to note that only three of the top 20 inhibitors of a parallel screen of the CHO-K1-Luc cell line that harbors a HTLV-1 LTR-luciferase reporter overlap with top hte 20 inhibitors of the TZM-bl screen (Table 2 and unpublished data). This indicates a high level of specificity of the lead inhibitors of either the HIV-1 or HTLV-1 provirus. Similarly, of the top 20 activators of the initial CHO-K1-Luc and TZM-bl screens, only four of the drugs overlapped (Table 1 and unpublished data), further supporting the hypothesis that the lead transcriptional modulators are highly specific to one proviral promoter or the other.

While the strategy we implemented to identify our lead hits is by no means the only method by which we could have screened the library of 420 FDA-approved drugs for transcriptional modulators, it has yielded promising drug candidates that work on HIV-1-infected cell lines and primary cells. Moreover, some of the drugs that were selectively eliminated due to their functional activation or inhibition of the CMV promoter in the second line of screening with the pc-Luc-transfected HeLa cells may in fact prove efficacious against the retroviruses of interest. This is due to the fact that the CMV promoter contains NF-κB enhancer elements [61,62,63], and similarly, there is a set of tandem NF-κB enhancer elements within the HIV-1 LTR [83]. Therefore, the lead HIV-1 transcriptional modulators from the initial screen that also down or upregulated the pc-Luc plasmid may in fact be valid inhibitors or activators of the HIV-1 provirus due to their ability to attenuate the NF-κB pathway. In order to address this potential inadvertent elimination of valid drug candidates, an alternate TZM-bl screen could be conducted with those drugs that were eliminated by transfecting with a Tat-expressing plasmid under the control of an alternate promoter that does not contain NF-κB enhancer elements or simply by using Tat protein. Despite the potential elimination of valid drug candidates, the benefit of removing drugs that altered the NF-κB pathway is that the manipulation of this important host cell pathway could be deleterious to infected patients. Additionally, this screen against the pc-Luc transfected cells also indicates the mechanism(s) of action for the remaining drugs carried forward likely does not include the overt activation or inhibition of the NF-κB pathway.

In our subsequent experiments, treating chronically infected cell lines with our lead compounds, the utility of our lead compounds in infected T cells was further demonstrated. Specifically, we showed that all three lead HIV-1 activators increased intracellular ENV copies in the latently infected ACH2 cell line (Figure 3A) and the pre-treatment of the ACH2 cells with ART for 11 days actually increased ENV copies from eltrombopag- and resveratrol-treated cells in this more patient-relevant setting (Figure 3B). Similarly, we observed substantial reductions in ENV copies from our treatment of the HIV-1-infected J1.1 cell line with mycophenolate (Figure 8A,B). As the primary read-out in these experiments was the direct measurement of full-length viral transcripts (both unspliced and singly spliced), the alteration in the copy number most likely demonstrates a direct impact of the experimental compounds on proviral transcription, although the enhanced stability or degradation of the mRNA cannot be ruled out for the activators and inhibitors, respectively.

In addition to testing chronically infected cell lines, an alternate reporter cell line was also examined with our lead hits. Specifically, the single-dose testing of the Jurkat E4 HIV-1 latency reporter cell line added supporting evidence of the efficacy and a low toxicity of all three HIV-1 activators (Figure 4). We were also able to leverage the short half-life GFP reporter signal in this model of latency to determine that the single dose efficacy of each of the lead activators was eliminated 72 h post-treatment (Figure 4B). Based on the loss of activity by 72 h, we tested multi-dose regimens of each of the activators in the Jurkat E4 cell line and found we could maintain the reporter GFP activation out to 72 h without increased toxicity (Figure 5). Moreover, the final concentration of the drug added in each of these multi-dose in vitro experiments is still lower than the plasma C_max_ observed in patients given a single dose of each of these drugs in clinical trials [71,72,73]. These data, therefore, substantiate the use of chronic multi-dosing regimens in future pre-clinical animal studies with the HIV-1 activators.

In a final test of efficacy, we also demonstrated that eltrombopag and mycophenolate can activate or inhibit transcription, respectively, in HIV-1-infected primary T cells that have been pre-treated with ART for 10 days (Figure 9A, upper panel). In this experiment, we found that eltrombopag increased ENV copies in three out of four infected donors, while mycophenolate decreased ENV copies in all three successfully tested donors (no RNA isolated from donor 2). This is the most relevant data described within this report that supports the continued examination of these drugs in HIV-1-infected humanized mouse models or non-human primates (NHPs).

We then utilized Tat mutants to better understand the function of our lead compounds. These studies showed that of the lead HIV-1 activators, febuxostat increased proviral transcription even in the absence of the Tat protein (Figure 10D), thereby demonstrating that its mechanism-of-action is not reliant on Tat-mediated transcription. Conversely, eltrombopag only appeared to activate the TZM-bl cell line in the presence of wild-type Tat (Figure 10A), indicating that it may require fully competent Tat-mediated transcription to elicit its activating effects. Alternately, the lead inhibitor, mycophenolate, was able to generate varied levels of suppression in the TZM-bl cells transfected with either wild-type Tat or the K51A mutant (Figure 10A,B) but did not significantly reduce the basal transcription in the K41A mutant and mock-transfected cells (Figure 10C,D). Therefore, mycophenolate may be useful in inhibiting Tat-mediated proviral transcription without impacting the basal transcriptional machinery of the host. Additionally, as previously mentioned, since the drugs that modulated the CMV promoter were eliminated from further testing in this study, it is likely that none of the remaining lead hits elicit their effects through the direct alteration of the NF-κB pathway.

Lastly, we carried out a Western blot analysis of the three lead HIV-1 proviral activators’ effects on several key host transcription factors, in both a T cell and myeloid lineage cell line. Here, we showed that eltrombopag increased the levels of p-Pol 2 (phospho-Ser2) and p-p65 (phospho-Ser536) at 24 h post-treatment in both the ACH2 and OM10.1 cell lines (Figure 12). Additionally, febuxostat treatment caused the upregulation of p-Pol 2 (phospho-Ser2) and p-p65 (phospho-Ser536) in only the ACH2 cell line and, conversely, resveratrol caused the upregulation of these two phosphorylated targets but only in the OM10.1 myeloid cell line (Figure 12). None of the experimental drugs caused changes in the p-TEFb component Cdk9, the bromodomain protein Brd4, nor the super elongation complex component AFF4. These results will help focus the future direction of studies of the potential MOA of these compounds to include the potential transient activation of the NF-κB pathway due to the increase in p-p65 by all three compounds in either one or both cell lineages. Additionally, it further punctuates the differential effects of some of the lead compounds (i.e., febuxostat and resveratrol) on cells of either T cell or myeloid lineage.

From previously published work, we can hypothesize on the mechanisms of action leading to the proviral transcriptional modulation observed here. With regard to the HIV-1 transcriptional activators, these three drugs have primary mechanisms of action that differ significantly. Specifically, febuxostat is a non-purine XO inhibitor used in the treatment of gout [64,65], eltrombopag is a TpoR agonist used to boost platelet counts in patients with idiopathic thrombocytopenia purpura (ITP) [66,84], and resveratrol is a potent antioxidant isolated from the *V. vinifera* grape that has been tested in numerous indications [67,85,86,87,88] The reduction of uric acid by febuxostat drives its efficacy in treating gout but as the enzymatic activity of XO also leads to the formation of reactive oxygen species (ROS), febuxostat can reduce oxidative stress in cells [89,90,91,92]. Interestingly, XO activity has been found to be 23% greater in HIV-1-infected patients on HAART [93]. Moreover, ROS produced by the addition of the exogenous XO-induced apoptosis of lymphocytes of all subsets from both HIV-1-infected and uninfected individuals [94]. Additionally, hyperuricemia is evident in HIV-1-infected patients with levels of uric acid increasing with disease progression [95]. Moreover, viral proteins including Tat and RT have been shown to increase ROS levels [96,97], but in contrast to our findings, most literature suggests that drugs that reduced ROS also inhibit proviral transcription [96,98]. Therefore, the mechanism by which febuxostat is enhancing proviral transcription is not readily evident. Our data, though, show that febuxostat actually works in a Tat-independent manner (Figure 10C,D) and it also was the top activator of the parallel CHO-K1-Luc HTLV-1 reporter screen (unpublished data). While it broadly activated both of these retroviruses, it did not activate the pc-Luc reporter construct that contains the CMV promoter (Figure 2A). In contrast to the finding that febuxostat did not activate the CMV promoter, we did observe an increase in p-p65 (phospho-Ser536) that indicates some level of activating the NF-κB pathway (Figure 12). Therefore, the activation of host transcription factors including NF-κB and the basal transcription machinery should be further explored to determine how this drug impacts proviral transcription. Additionally, beyond the activation of proviral transcription observed here, febuxostat could protect uninfected lymphocytes and other bystander cells within infected individuals from the increased ROS burden found in HIV-1 patients. The reduced ROS burden could help to maintain the integrity of patients’ immune system and maintain the health of other tissues, as well as minimize the release of virions from apoptotic infected cells.

As for the TpoR agonist eltrombopag, the activation of its target receptor results in the activation of the Janus kinase 2 (JAK2) [99] and its yields varied downstream signal transduction activation including AKT [100], STAT 1, 3 and 5 [100], as well as Erk 1/2 [101,102,103]. Observed downstream activation by the TpoR natural ligand, thrombopoietin (Tpo), does differ from that observed with eltrombopag with AKT phosphorylation lacking in eltrombopag-treated cultures, but Stat 1, 3, and 5 activation was observed with both molecules [100]. Retained Stat activation is important to latent HIV-1 reactivation as a recent study has shown that the small molecule inhibition of Stats blocks HIV-1 reactivation by three other LRAs, specifically SAHA, prostratin, and TNFα [104]. Furthermore, Erk 1/2 activation by TCR stimulation has also been shown to activate P-TEFb, which is critical for the efficient transcriptional elongation of the provirus [29]. Additionally, Tpo stimulation has also been shown to transiently increase IKK activity and downstream NF-κB signaling followed by a significant decrease in IKK kinase activity over time in a megakaryocyte cell line [103]. This abbreviated temporal activation of NF-κB could explain why eltrombopag has a comparable or better activation of proviral transcription than febuxostat in ACH2 and Jurkat E4 cells (Figure 3A,B, Figure 4A,B, and Figure 5A,B,D), despite febuxostat showing greater activation in the TZM-bl screen (Figure 1A,B). Specifically, the initial burst of NF-κB activation could produce enough of the Tat protein in the Tat-producing ACH2 and Jurkat E4 cell lines to sustain subsequent Tat-mediated transcription. Conversely, the TZM-bl cell line does not contain an integrated Tat gene under the control of the viral promoter and therefore, cannot elicit positive Tat-feedback transcriptional activation. A short burst of NF-κB activation may also not be sufficient to elevate luciferase levels in the pc-Luc screening assay which would have eliminated eltrombopag as a potential false positive hit in the initial TZM-bl screen (Figure 2A). Our Western blot analysis also detected an increase in p-p65 (phospho-Ser536) at 24 h post-treatment (Figure 12), further validating the potential of eltrombopag to elicit a temporal burst of NF-κB activation. Additional examination of IKK and NF-κB activation kinetics, as well as Tat expression levels in those cells containing an integrated Tat gene need to be carried out to confirm this hypothesis.

In addition to the cellular and biochemical study of eltrombopag, clinical trials have already been conducted for examining pharmacokenetics/pharmacodynamics (PKPD) in healthy subjects co-administered eltrombopag and a lopinavir/ritonovir (LPV/RTV) HAART regimen [105]. This clinical study only showed a modest 17% reduction in the average eltrombopag serum levels and no change in the LPV/RTV levels, indicating the bioavailability of both eltrombopag and LPV/RTV in a potential co-administration regimen. As this trial was only conducted in uninfected subjects to demonstrate the PKPD of eltrombopag added to patients already on HAART that are also diagnosed with ITP, neither viral RNA copies nor integrated proviral DNA levels were tested in this clinical trial. Nonetheless, this study is a key first step for subsequently testing the efficacy of eltrombopag in reducing the latent reservoir in Phase 2 clinical trials.

The last of our lead LRAs, the antioxidant resveratrol, has been tested in numerous indications including both viral and bacterial infections [85,86], various cancers [89], and metabolic syndromes [88]. It has also been previously identified as a potential LRA for reactivating HIV [68]. In regards to HIV, resveratrol activation of the protein deacetylase Sirtuin 1 (SIRT1) [106] and AMP-activated protein kinase (AMPK) [107] could explain the enhanced proviral transcription observed here. SIRT1 has been shown to be requisite in Tat-mediated transcription as the inhibition of SIRT1 by siRNA or small molecule inhibitors ablated Tat-mediated transcription [108]. The activation of HIV-1 LTR by SIRT1 in the presence of Tat was independent of NF-κB activity, consistent with the findings in our pc-Luc screen which showed no activation of the NF-κB enhancer element containing plasmid by resveratrol (Figure 2A). The resveratrol activation of AMPK is equally important as AMPK is a master regulator of several downstream factors key to proviral transcription including Protein kinase C–θ (PKCθ) [109]. PKCθ activity has been shown to be integral to the mechanism of action of several well studied LRAs, as a small molecule inhibitor of PKCθ blocks HIV-1 reactivation by SAHA, prostratin, and TNFα [104]. Therefore, the further examination of the SIRT1 and AMPK activation by resveratrol is warranted to determine if these pathways are indeed the molecular MOA leading to the enhanced proviral transcription observed here.

As for the lead HIV-1 transcriptional inhibitor, mycophenolate, its primary mechanism of action is to block the de novo synthesis of guanosine through the inhibition of inosine-5′-monophosphate dehydrogenase [110,111]). Moreover, mycophenolate and its prodrug mycophenolate mefotil have already been shown to inhibit the replication of HIV-1 both in vitro and in vivo [76,112], and was a component of the immunosuppressant therapeutic regimen of the only patient known to be cured of his HIV-1 infection [113]. In regards to the MOA, most studies have focused on the ability of mycophenolate to suppress activated T cell proliferation [112,114]. To date though, no studies documenting the inhibition of proviral transcription by mycophenolate or mycophenolate mefotil have been conducted; therefore, this work is the first to demonstrate this basic mechanism of action of mycophenolate on HIV-1-infected cells.

Another important observation from this study was that significant differences in response to experimental treatment were noted between those in infected T cells and cells of myeloid lineage. This is not the first time that such a difference in response to LRAs has been observed between primary T cells and myeloidal cells. Specifically, HDACis are used to activate latent provirus in quiescent CD4+ T cells, which actually suppresses the viral production from macrophages due to the activation of autophagy [38]. Here, we observed that while our lead HIV-1 activators did in fact increase ENV copies from the latently HIV-1-infected ACH2 T cell line, we actually saw no comparable increase in ENV transcripts in the latently HIV-1-infected OM10.1 promyelocytic cell line. Similarly, while our lead HIV-1 inhibitor, mycophenolate, did indeed reduce ENV copies in the productively HIV-1-infected J1.1 T cell line, it actually increased ENV copies when used to treat the promyelocytic OM10.1 cells. More importantly, while eltrombopag and mycophenolate successfully increased and decreased ENV copies in infected primary T cells, we observed no corresponding modulation in the infected primary MDMs. These observations warrant the further examination of the fundamental differences in transcription and viral production from these two important host reservoirs of HIV-1 provirus. Furthermore, the differing responses of these two infected cell types to the same drug suggests that the development of unique therapeutic interventions to each will likely be required to elicit functional or sterilizing elimination of the provirus from these two cell lineages.

In conclusion, here, we narrowed down a library of 420 FDA-approved drugs using a sequence of three assays to identify three HIV-1 transcriptional activators and one HIV-1 transcriptional inhibitor. All of these drugs then demonstrated varying levels of transcriptional modulation in the subsequent testing of infected cell lines and alternate reporter assays. Moreover, our lead HIV-1 activator, eltrombopag, and HIV-1 inhibitor, mycophenolate, showed increases and decreases in viral ENV copies, respectively, in infected primary T cells. Additionally, we were able to begin narrowing down the potential mechanism(s) of action of the lead transcriptional modulators through the use of functional mutants of the HIV-1 transcriptional transactivating protein, Tat. Going forward, fundamental questions generated in this study, such as the divergent effects of the lead compounds on cells of lymphoid and myeloid origin, must be addressed. Moreover, demonstrating the efficacy of these promising lead compounds in HIV-1-infected humanized mouse models or NHPs is required in order to proceed to human clinical trials.

## Figures and Tables

**Table 1:** List of the top 20 activators of TZM-bl drug screen. Ranked listing of the top 20 FDA-approved drugs screened against pcTat-activated TZM-bl reporter cells. The average luminescence reading of the DMSO controls was set to 100% and all experimental readings were normalized to the DMSO control. Brief descriptions of the known molecular mechanism and/or current indication of each drug are given in the right column. The red box indicates the top seven activators in the initial reporter cell line screen.

Drug Name	% Luminescence of pcTat + DMSO	Brief Description
Febuxostat (Uloric)	459.50	Febuxostat is a non-purine selective xanthine oxidase inhibitor with an IC50 of 114–210 nM.
Nitrofurazone (Nitrofural)	271.89	Nitrofurazone is a topical anti-infective agent with an IC50 of 22.83 ± 1.2 µM. (Rat LD50 = 590 mg/kg).
Nitazoxanide (Alinia, Annita)	261.92	Nitazoxanide (Alinia, Annita) is a synthetic nitrothiazolyl-salicylamide derivative and an antiprotozoal agent. (IC50 for canine influenza virus ranges from 0.17 to 0.21 μM).
Eltrombopag (SB-497115-GR)	205.50	Eltrombopag (SB-497115-GR, Promacta, Revolade) is a small molecule agonist of the c-mpl (TpoR) receptor with an IC50 of 0.69 μM for the inhibition of hERG K+ channel tail current.
Vincristine	170.52	Vincristine sulfate is a microtubule function inhibitor with an IC50 of 10.4 ± 1.1, 28.1 ± 3.4, 22.4 ± 2.1 µM for HL-60, Bel7402, HO-8910, respectively.
Resveratrol	168.85	Resveratrol is a phytoalexin produced naturally by several plants with anti-cancer, anti-inflammatory, blood-sugar-lowering and other beneficial cardiovascular effects.
Prednisolone acetate (Omnipred)	168.32	Prednisolone acetate is a synthetic corticosteroid drug that is particularly effective as an immunosuppressant agent.
Ethinyl estradiol	157.10	Ethynyl estradiol is an orally bio-active estrogen used in almost all modern formulations of combined oral contraceptive pills.
Econazole nitrate (Spectazole)	156.49	Econazole nitrate (Spectazole) is an imidazole class antifungal medication.
Budesonide	154.59	Budesonide is a glucocorticoid steroid for the treatment of asthma, non-infectious rhinitis.
Aztreonam (Azactam, Cayston)	152.67	Aztreonam (Azactam, Cayston) is a synthetic monocyclic beta-lactam antibiotic.
Vorinostat (SAHA)	150.70	Vorinostat also known as SAHA, Zolinza, MK-0683 is an HDAC inhibitor. Vorinostat CAS No 149647-78-9 with purity >99% and solubility DMSO is available.
Omeprazole (Prilosec)	147.83	Omeprazole (Prilosec) is a proton pump inhibitor used in the treatment of dyspepsia.
Minoxidil	144.63	Minoxidil is a vasodilator medication known for its ability to slow or stop hair loss and promote hair regrowth.
Clorsulon	139.45	Clorsulon is a competitive 8-phosphoglycerate kinase and phospho-glyceromutase inhibitor.
Leflunomide	139.23	Immunosuppressant agent. Its active metabolite is A77 1726 (RS-61980).
Amorolfine hydrochloride	137.65	Amorolfine hydrochloride is an antifungal reagent.
Azelastine hydrochloride (Astelin)	137.36	Azelastine is a potent, second-generation, selective, histamine antagonist.
Perindopril erbumine (Aceon)	136.11	Perindopril erbumine(Aceon) is the tert-butylamine salt of perindopril, the ethyl ester of a non-sulfhydryl angiotensin-converting enzyme (ACE) inhibitor.
Fenoprofen calcium	135.40	Fenoprofen calcium is a nonsteroidal, anti-inflammatory antiarthritic agent.

**Table 2:** List of the top 20 inhibitors of TZM-bl drug screen. Ranked listing of the top 20 FDA-approved drugs screened against pcTat-activated TZM-bl reporter cells. The average luminescence reading of the DMSO controls was set to 100% and all experimental readings were normalized to the DMSO control. A brief description of the known molecular mechanism and/or current indication of each drug is given in the right column. The red box indicates the top seven inhibitors in the initial reporter cell line screen.

Drug Name	% Luminescence of pcTat + DMSO	Brief Description
Idarubicin HCl	2.58	Idarubicin is the anthracycline antibiotic and target DNA topoisomerase II (topo II). MCF-7 cells were sensitive to idarubicin, with an IC50 value for growth inhibition of 0.01 μM.
Bortezomib (Velcade)	2.81	Bortezomib also known as Velcade, MG-341, PS-341 is a proteasome inhibitor, effectively inhibits proteasome activity (Ki-0.6 nM).
Camptothecin	11.15	Camptothecin (CPT) is a cytotoxic quinoline alkaloid which inhibits the DNA enzyme topoisomerase I (topo I) with an IC50 and IC70 of 50 nM and 0. 225 μM.
Topotecan HCl	11.27	Topotecan hydrochloride (Hycamtin) is a topoisomerase I inhibitor with an IC50 of 13 and 2 nM for MCF-7 Luc cells and DU-145 Luc cells.
Mitoxantrone hydrochloride	20.59	Mitoxantrone is a type II topoisomerase inhibitor with an IC50 of 2.0 µM, 0.42 mM for HepG2 and MCF-7/wt cells, respectively.
Cladribine	22.74	Cladribine (Leustatin) is an adenosine deaminase inhibitor with an IC50 of about 0.2 μM.
Mycophenolic (mycophenolate)	24.00	Mycophenolic acid (Mycophenolate) is an immunosuppressant drug used to prevent rejection in organ transplantation.
Gemcitabine HCl (Gemzar)	27.23	Gemcitabine is an antimetabolite-inhibiting DNA synthesis (cell IC50 of 0.06 µM).
Floxuridine	27.41	Floxuridine (FUDR, FdUrd, Floxuridin) is a pro-drug of floxuridine and an oncology drug with an GI50 of 5.1 μM for the inhibition of MDCK/PEPT1.
Clofarabine	28.18	Inhibits the enzymatic activities of ribonucleotide reductase (IC50 = 65 nM) and DNA polymerase
Epirubicin hydrochloride	30.77	Epirubicin hydrochloride (Ellence) is an anthracyclin antibiotic and a DNA topoisomerase II inhibitor with an IC50 of 12 μg/mL on an estrogen-receptor-positive ER (+) hyperdiploid EAT cell line.
Sorafenib (Nexavar)	31.46	Sorafenib Tosylate is a novel, small molecular inhibitor of several tyrosine protein kinases (VEGFR and PDGFR) and a RAF/MEK/ERK cascade inhibitor with an IC50 of 6, 22, 38 nM for Raf-1, wt BRAF and V599E mutant BRAF.
Doxorubicin (Adriamycin)	32.46	Doxorubicin is a topoisomerase II inhibitor with an IC50 of 1 and 2 μM for the inhibition of MCF-7 and MDA-MB231.
Pemetrexed	34.97	Pemetrexed disodium (Alimta) is a thymidylate synthase, DHFR and glycinamideribonucleotide, and formyltransferase inhibitor with IC50s of 25, 34, 220 nM, respectively.
Mycophenolate mofetil (CellCept)	36.45	Mycophenolate mofetil is an inhibitor of inosine monophosphate dehydrogenase and an immunosuppressant.
Axitinib	36.49	Axitinib blocks the phosphorylation of VEGFR-2 and VEGFR-3 with average IC50s of 0.2 and 0.1 to 0.3 nM.
Teniposide (Vumon)	36.85	Teniposide (Vumon) is a chemotherapeutic medication mainly used in the treatment of childhood acute lymphocytic leukemia (ALL).
Tretinoin (Aberela)	46.89	Tretinoin (Aberela) is a drug commonly used to treat acne vulgaris and keratosis pilaris.
Sunitinib malate (Sutent)	49.15	Sunitinib malate (Sutent) is a multitargeted FLT3, PDGFRs, VEGFRs, and Kit kinase inhibitor with a Ki of 0.009 and 0.008 µM for Flk-1 and PDGFR.
Nilotinib (AMN-107)	49.44	Inhibitor of BCR-ABL, IC50 < 30 nM
